# Entry of Human Papillomavirus Type 16 by Actin-Dependent, Clathrin- and Lipid Raft-Independent Endocytosis

**DOI:** 10.1371/journal.ppat.1002657

**Published:** 2012-04-19

**Authors:** Mario Schelhaas, Bhavin Shah, Michael Holzer, Peter Blattmann, Lena Kühling, Patricia M. Day, John T. Schiller, Ari Helenius

**Affiliations:** 1 Emmy-Noether Group ‚Virus Endocytosis', Institutes of Molecular Virology and Medical Biochemistry, University of Münster, Münster, Germany; 2 Institute of Biochemistry, ETH Zurich, Zurich, Switzerland; 3 Laboratory of Cellular Oncology, National Cancer Institute, National Institutes of Health, Bethesda, Maryland, United States of America; Penn State University School of Medicine, United States of America

## Abstract

Infectious endocytosis of incoming human papillomavirus type 16 (HPV-16), the main etiological agent of cervical cancer, is poorly characterized in terms of cellular requirements and pathways. Conflicting reports attribute HPV-16 entry to clathrin-dependent and -independent mechanisms. To comprehensively describe the cell biological features of HPV-16 entry into human epithelial cells, we compared HPV-16 pseudovirion (PsV) infection in the context of cell perturbations (drug inhibition, siRNA silencing, overexpression of dominant mutants) to five other viruses (influenza A virus, Semliki Forest virus, simian virus 40, vesicular stomatitis virus, and vaccinia virus) with defined endocytic requirements. Our analysis included infection data, i.e. GFP expression after plasmid delivery by HPV-16 PsV, and endocytosis assays in combination with electron, immunofluorescence, and video microscopy. The results indicated that HPV-16 entry into HeLa and HaCaT cells was clathrin-, caveolin-, cholesterol- and dynamin-independent. The virus made use of a potentially novel ligand-induced endocytic pathway related to macropinocytosis. This pathway was distinct from classical macropinocytosis in regards to vesicle size, cholesterol-sensitivity, and GTPase requirements, but similar in respect to the need for tyrosine kinase signaling, actin dynamics, Na^+^/H^+^ exchangers, PAK-1 and PKC. After internalization the virus was transported to late endosomes and/or endolysosomes, and activated through exposure to low pH.

## Author Summary

Human papillomavirus type 16 is the main etiological agent of cervical cancer. Despite advances in our understanding of transformation and cancer progression, as well as preventative vaccination strategies, the early events in papillomavirus infections are incompletely understood. Here, we investigated which strategies and cellular mechanisms the virus uses to enter epithelial cells. Entry was slow and asynchronous likely due to several structural alterations, which needed to occur on the cell exterior. Interestingly, the virus hijacked a potentially novel pathway of endocytosis for entry, which was distinct from classical macropinocytosis in regards to vesicle size, cholesterol-sensitivity, and GTPase requirements, but similar in respect to tyrosine kinase signaling, actin dynamics, Na^+^/H^+^ exchangers, PAK-1 and PKC requirements. This cellular mechanism may also be used by other viruses such as influenza A virus, echo virus 1, and choriomeningitis virus.

## Introduction

Human Papillomaviruses (HPVs) are a family of small non-enveloped viruses that induce mostly benign papillomas. However, several high risk HPV types, most prominently HPV-16, cause cervical cancer and other epithelial tumors. While the molecular biology of transformation has been studied in some detail [1], the cell biology of HPV-16 entry is still a subject of scientific debate [2]. Transmission is conferred by virions that contain two structural proteins, L1 and L2. These provide an icosahedral (T = 7) capsid of 52–55 nm that protects the closed, circular, double-stranded DNA genome. HPVs initially enter basal cells of stratified epithelia [3]. The productive life cycle of HPVs requires differentiating human epidermal tissue allowing only limited infectious virus production *in vitro*. As a surrogate model, entry into a variety of cell lines has been studied using HPV virus-like particles (VLP) or pseudovirions (PsV) [4]–[9], i.e. viral capsids harbouring a reporter plasmid [10].

HPV-16 can bind to cell surface receptors and to the extracellular matrix (ECM). Binding to cells occurs through interaction with glycosaminoglycans (GAGs), mostly heparan sulfate proteoglycans (HSPGs, [11]–[14]). Binding to the ECM may additionally involve laminin-332 [15]. Binding results in conformational changes in the capsid that allow partial externalization of the inner virion protein L2 by cyclophilin B. This, in turn, leads to cleavage of the N-terminus proximal sequence of L2 by furin [16]–[20]. Transfer from HSPGs to a putative secondary receptor has been proposed to precede infectious internalization [21].

The literature on papillomavirus entry does not allow a generalized view regarding the mode of endocytosis. Several studies with HPV-16, HPV-31, and BPV-1 have relied on the small compound inhibitor chlorpromazine to suggest entry by clathrin-mediated endocytosis (CME) [5], [6], [22]. In addition, dynamin-2 has been suggested as a mediator in primary endocytic vesicle (PEV) formation for HPV-16 [4]. However, recent studies challenge an unambiguous role of CME in entry of papillomaviruses: HPV-31 entry into keratinocytes has been attributed to caveolar/lipid-raft mediated endocytosis [5], [8], and Spoden and colleagues [9] suggested that HPV-16 entry into 293TT and HeLa cells occurs through tetraspanin-enriched microdomains, and is clathrin-, lipid raft- and dynamin-independent. As a minimal consensus, all studies agree that HPV-16 entry is insensitive to cholesterol depletion, but sensitive to lysosomotropic agents. Some of the confusion may be explained by the use different VLP/PsV preparations and cell lines.

In this study, we addressed HPV-16 entry by systematically perturbing the function of numerous cellular key factors implicated in the various endocytic mechanisms known to date using chemical inhibitors, siRNA silencing, and overexpression of dominant negative (DN) proteins. Immunofluorescence analysis, live cell imaging, and thin section electron microscopy were used to analyze viral colocalization or cotrafficking with cellular factors, and to visualize the morphology of virus-containing vesicles. To circumvent some limitations that cellular endocytic ligands may pose for a comparative analysis such as monovalency, lesser or higher sensitivity to pertubations, different time courses, etc., we used viruses with known endocytic requirements as controls, i.e. simian virus 40 (SV40), Semliki Forest virus (SFV), influenza A virus (IAV), vesicular stomatitis virus (VSV), and vaccinia virus (VV). The results indicated that HPV-16 entry was clathrin-, caveolin-, flotillin-, cholesterol-, and dynamin-independent and did not involve the glycosphingolipid enriched endocytic carrier (GEEC) pathway, the Arf6 pathway, and the IL-2 pathway. In contrast, HPV-16 made use of a potentially novel endocytic pathway possibly related to macropinocytosis, which was dependent on actin dynamics and tyrosine kinase signaling. The viruses were carried to late endosomes/lysosomes and underwent slow acid-dependent penetration.

## Results

### Kinetics of HPV-16 endocytosis

First, we analyzed how rapidly infectious cell-bound HPV-16 particles were internalized by endocytosis in HeLa and HaCaT cells. To distinguish between external and internalized viruses, we used a high pH wash to inactivate the PsV located on the cell surface (see Figure S1 A, B, and material and methods for details). HPV-16 PsV were bound to cells in the cold, unbound virus was washed away, and the cells were warmed to 37°C. At different times post warming, cells were subjected briefly to a high pH wash and infection was scored after 48 h by the detection of GFP expression from the PsV reporter plasmid using flow cytometry.

This way the internalization exclusively of infectious particles was probed.

**Figure 1:**
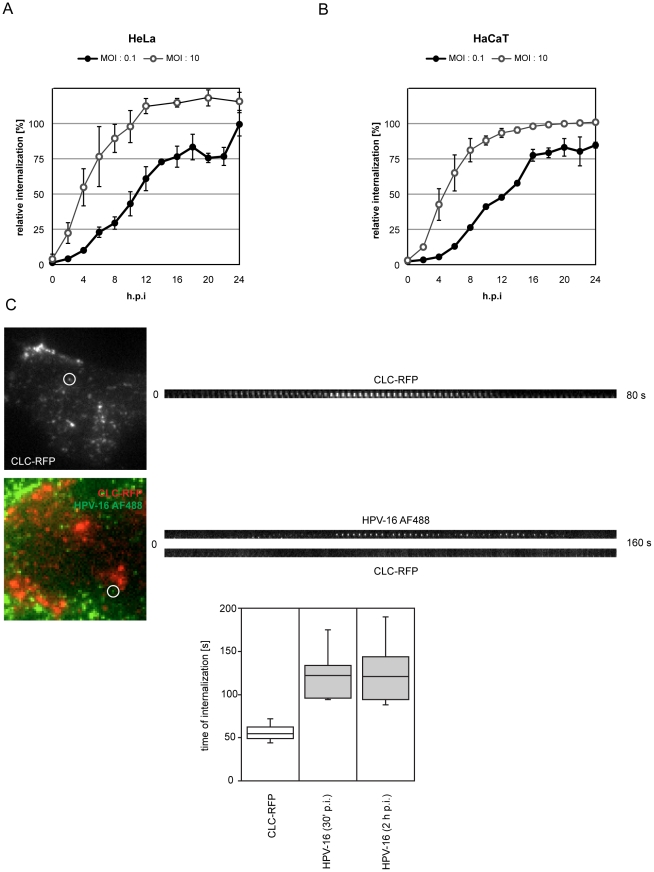
HPV-16 internalization events are fast and occur asynchronously over several hours. To test the average internalization kinetics of infectious HPV-16 PsV, HeLa (A) or HaCaT (B) cells were infected with HPV-16 PsV at MOIs of 0.1 vs. 10 transducing units/cell, i.e. about 10 vs. 1,000 particles/cell (black vs. white circles, respectively). Virus was bound to cells at 4°C, warmed to 37°C and at indicated times post warming, i.e. post infection (p.i.), cells were treated with pH 10.5 for 1 min. to inactivate extracellular virus. 48 h p.i. cells were analyzed for GFP expression from the PsV reporter plasmid by flow cytometry. The amount of GFP expressing cells relative to untreated control is given as relative internalization in %. (C) To test the internalization kinetics of single particles, we infected HeLa cells with HPV-16 PsV labeled with AF488 (5,000 particles/cell, HPV-16 AF488, green). Cells were transfected 16 h prior to infection with a construct expressing clathrin light chain-mRFP (red) to investigate any potential cointernalization. Depicted are TIRFM images of an uninfected (**C**, above) and infected (**C**, below) cell. Kymographs to the right of the images depict the formation and internalization of a CCP or virus (encircled in the images, also shown in Video S1), respectively. Internalization was defined as the loss of fluorescent signal. The time span required for CCP formation or virus internalization was determined from the initial detection of fluorescence to complete loss of fluorescence for CCPs, or from the moment of confinement to loss of fluorescence for HPV-16, respectively. The results were quantified manually and plotted (**C**, far below).

When using a low multiplicity of infection (MOI, 0.1 tdu/cell), so that a single infectious particle per cell caused infection, we found that about 2 h at 37°C were needed for the first viruses to be internalized. The half-time for the entire population was 11 h (Figure 1A, black). When a higher MOI (10 tdu/cell) was used, infectious internalization by the first (fastest) of several infectious particles caused infection providing information regarding the fastest possible internalization kinetics. The fastest particles provided a half time of internalization of 4 h post warming with no apparent lag time (Figure 1A, white). Similar internalization kinetics were observed in HaCaT cells (Figure 1B).

When internalization of fluorescently labeled HPV-16 particles was analyzed by scoring cell-associated fluorescence after removing cell surface-bound viruses by protease digestion at different times post warming, similar kinetics as compared to the high MOI infectious internalization were observed (data not shown). This suggested that all particles, infectious and non-infectious, followed a similar itinerary.

With half times of internalization ranging from 4–12 h p.i. for the fastest and average particles, our data was consistent with previous observations, where bulk internalization of papillomaviruses and PsV was shown to be extremely slow [6], [23], [24]. To our knowledge, this describes the slowest internalization kinetics observed for a virus.

Endocytic internalization of individual viruses in live cells can be visualized using total internal reflection microscopy (TIRFM) by the loss of fluorescence as vesicles leave the evanescent field [25]. We used fluorophore labeled PsV that can be easily followed by microscopy and retain their infectivity after labelling [7]. As papillomavirus entry has been mostly attributed to CME, we analyzed internalization of AF488-labeled virions in cells expressing clathrin light chain tagged with mRFP (CLC-RFP) to follow any potential cointernalization.

After binding to cells, HPV-16 particles exhibit periods of lateral diffusion and directed transport, after which movement stops [7]. Such confinement is often associated with the interaction of plasma membrane receptors with the actin cytoskeleton or endocytic coat elements [26]. We analyzed how quickly the fluorescence of HPV-16 PsV particles was lost after confinement, an event consistent with internalization. The majority of particles stayed confined and were not internalized during data acquisition possibly due to HPV-16 anchoring to ECM factors and plasma membrane receptors [15], [27]. However, internalization of several particles (30 min p.i., n = 17; 2 h p.i., n = 31) could be observed, and when that happened it occurred on average within 120 s post confinement (Figure 1C, Video S1). The fastest event occurred 93 s after confinement. The formation and endocytosis of clathrin coated pits was readily observed by TIRFM in HeLa cells. Consistent with previous reports, the full cycle lasted about 60 s (Figure 1C) [28], [29]. Cointernalization of HPV-16 and clathrin was not observed. Thus we found that single virus internalization events were fast, but occurred only sporadically over a period of many hours. The absence of any clathrin signal during internalization suggested a clathrin-independent endocytosis event.

### Analyzing HPV-16 entry by perturbations

This prompted us to reinvestigate the endocytic requirements of HPV-16 entry. We used small compound inhibitors, siRNA-mediated depletion, and overexpression of DN proteins to identify the cellular factors required or dispensable for infectious endocytosis.

To reduce potential cytotoxic effects of cellular inhibitors, the inhibitors were replaced after the entry phase was over by a less toxic inhibitor such as NH_4_Cl that blocked acid-activation of viruses in endosomes (Figure S2A). Swapping for a second inhibitor allowed us to assess the effect of an inhibitor of interest on pre-acidification in the entry process such as binding, endocytosis and perhaps intracellular routing (compare Figure S2A, Table S1, and material and methods). The infection data was normalized to cells in which only the secondary inhibitor was used. In addition, we excluded any data that exhibited significant cytotoxic effects, i.e. a reduction of cell number by more than 50% for a given pertubation. Infection and cytotoxicity was analyzed by automated microscopy and computational analysis of cell number and infection (see Figure S2B and material and methods for details).

To minimize erroneous interpretation of data, and to exclude pleiotrophic effects beyond cell toxicity, we controlled for the specificity and efficiency of inhibition using prototype virus infection experiments using the same inhibitor. In addition, cell biological controls such as transferrin uptake for CME were more sensitive to pertubation than virus infection (data not shown). Here, we used SFV and VSV as controls entering by CME, and SV40 using caveolar/lipid-raft dependent endocytosis. Further controls included infection by VV for macropinocytosis, and IAV for penetration of late endosomal compartments. To stay within one experimental system for comparison of the different viruses, we used HeLa cells but verified key findings for HPV-16 in HaCaT cells. Since the dominant effect of mutant proteins often depends on the level of overexpression, we analyzed infection of transfected cells by flow cytometry and grouped the data into low, medium, and high mutant protein expressing cells (Figure S2C).

### HPV-16 entry is clathrin-, caveolin-, and flotillin-independent

**Figure 2:**
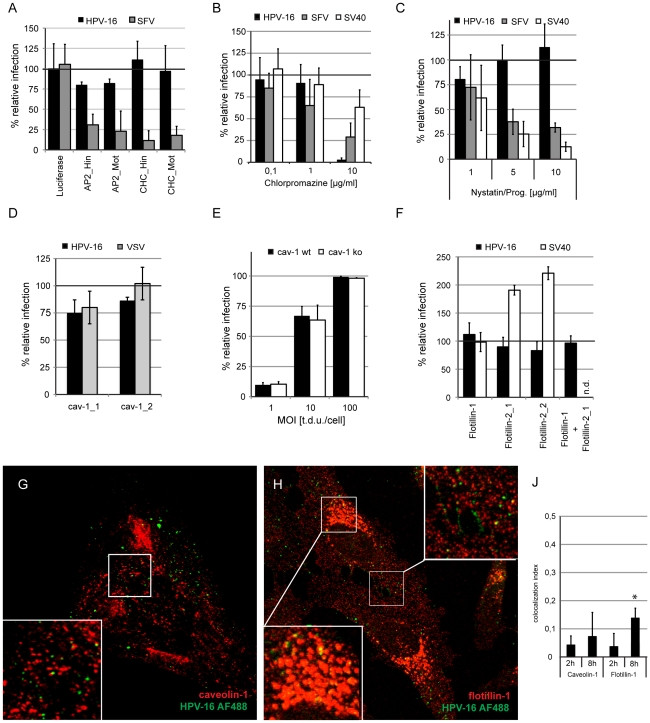
HPV-16 entry is clathrin-, caveolin-, cholesterol- and flotillin-independent. (A) HeLa cells were transfected with siRNA oligos directed against the AP2μ-subunit (AP2), clathrin heavy chain (CHC), or as control against luciferase. Cells were infected with HPV-16 PsV or SFV and infection was scored 24 or 6 h p.i. by flow cytometry, respectively. Depicted are the mean infection percentages relative to the luciferase control. (B) HeLa cells were pretreated for 30 min with chlorpromazine in the indicated concentrations. Cells were infected with HPV-16 PsV (black), SFV (grey), or SV40 (white) in the presence of inhibitor. Inhibitors were exchanged for HPV-16 and SV40 with NH_4_Cl or DTT, respectively. Infection was scored by automated microscopy and image analysis. Depicted are results normalized to solvent treated control cells. See also Figure S2, and material and methods. (C) HeLa cells were pretreated overnight with nystatin/progesterone in the indicated concentrations. Cells were infected with HPV-16 PsV (black), SFV (grey), or SV40 (white) and analyzed as in (B). (D) HeLa cells were transfected with siRNA oligos directed against caveolin-1 or with the AllStar negative control (Qiagen). 48 h after siRNA transfection cells were infected with HPV-16 PsV (black) or VSV (grey). Infection was scored as in (B) and depicted relative to cells transfected with the AllStar negative control. (E) Mouse embryonic fibroblasts from caveolin-1 knockout mice or parental mice were infected with HPV-16 PsV at different MOI. Infection was scored by flow cytometry as described in material and methods. Depicted are infection percentages. (F) HeLa cells were transfected with siRNA oligos directed against flotillin-1 or −2 or with the AllStar negative control (Qiagen). 48 h after siRNA transfection cells were infected with HPV-16 PsV (black) or SV40 (white). Infection was scored as in (C) and depicted relative to cells transfected with the AllStar negative control. (G, H) Merge images of a single cLSM slice of caveolin-1 (J, red) or flotillin-1 (K, red) HPV-16 AF488 (green) fluorescence of HeLa cells infected for 8 h. Boxed regions were enlarged as insets. (J) HeLa cells were infected with HPV-16 AF488 for 2 or 8 h, fixed with paraformaldehyde and immunostained for caveolin-1 or flotillin-1. Samples were analyzed by confocal laser scanning microscopy (cLSM) and the colocalization indices were determined as described in material and methods. Asterisk indicates a significant Costes P-value.

First, we analyzed whether infection of HPV-16 in HeLa cells was clathrin-dependent. To inhibit CME, we used siRNA to deplete the clathrin heavy chain or the μ subunit of the clathrin adaptor AP2. A depletion to 20% or less of the protein levels in control cells resulted in efficient decrease in SFV infection (Figure 2A, Figure S3A), a virus that enters exclusively by CME [30]–[32]. HPV-16 infection was not significantly affected (Figure 2A), indicating that HPV-16 entry was clathrin- and AP2-independent. This was consistent with the TIRFM results described above.

Chlorpromazine, generally considered an inhibitor of CME, abolished HPV-16 infection at 10 µg/ml in line with earlier studies [5], [8], [22]. At this concentration, chlorpromazine also partially affected infection of SV40, a virus that makes use of CME-independent endocytosis (Figure 2B). Since efficient clathrin and AP2 depletion did not affect HPV-16 infection, we suspected chlorpromazine to cause CME-independent inhibition of entry.

To test whether HPV-16 was using a caveolin/lipid raft-mediated pathway, we depleted cells of cholesterol with nystatin/progesterone, a combination of drugs often used to inhibit fomation of lipid rafts in the plasma membrane [33]. SV40 infection, which uses a combination of a caveolar and a caveolin-independent lipid raft-mediated mechanism [34]–[36], was efficiently inhibited (Figure 2C). SFV infection was also affected most likely due to its cholesterol-dependent membrane fusion activity [37]. However, HPV-16 entry was, if anything, increased (Figure 2D). Similarly, cholesterol extraction by methyl-ß-cyclodextrin had only little effect (Table S1). We found, moreover, that HPV-16 infected HeLa cells depleted of caveolin-1 and caveolin-1 knockout cells with the same efficiency as wild-type cells (Figure 2D, E, Figure S3B). Since SV40 can make use of a caveolin-independent lipid-raft dependent pathway [34], and since cholera toxin B, that can enter cells by caveolar endocytosis, can make use of multiple pathways [38], meaningful positive controls could unfortunately not be employed.

To test whether endocytosis of HPV-16 would involve flotillins, proposed to define a distinct endocytic mechanisms [39], we depleted cells of flotillin-1 or -2, or both by siRNA mediated knockdown. Since no viral cargo exists for flotillin endocytosis, and since cholera toxin B can use multiple pathways, again, no direct positive controls could be used. Independent or combined depletion of flotillin-1 and -2 to 20% protein levels or less of control cells had no effect on HPV-16 infection, whereas SV40 infection was enhanced after flotillin-2 but not -1 knockdown (Figure 2F, Figure S3C).

We observed no significant colocalization of HPV-16 with caveolin-1 or flotillin-1 at 2 h post warming by confocal microscopy (Figure 2G–J). At 8 h, the degree of colocalization between HPV-16 and caveolin or flotillin increased slightly (Figure 2H–J). The colocalizing objects were mostly located intracellularly and perinuclearly, which suggested that they belonged to a population of endosomal vacuoles (Fig. 2G, H; [35], [40]).

In summary, infectious HPV-16 entry was independent of clathrin, AP2, caveolin, lipid rafts and flotillin, and HPV-16 did not associate with caveolin or flotillin at the cell surface. This indicated that HPV-16 entry occurred by a distinct endocytic mechanism.

### HPV-16 entry is dynamin-2 independent

**Figure 3:**
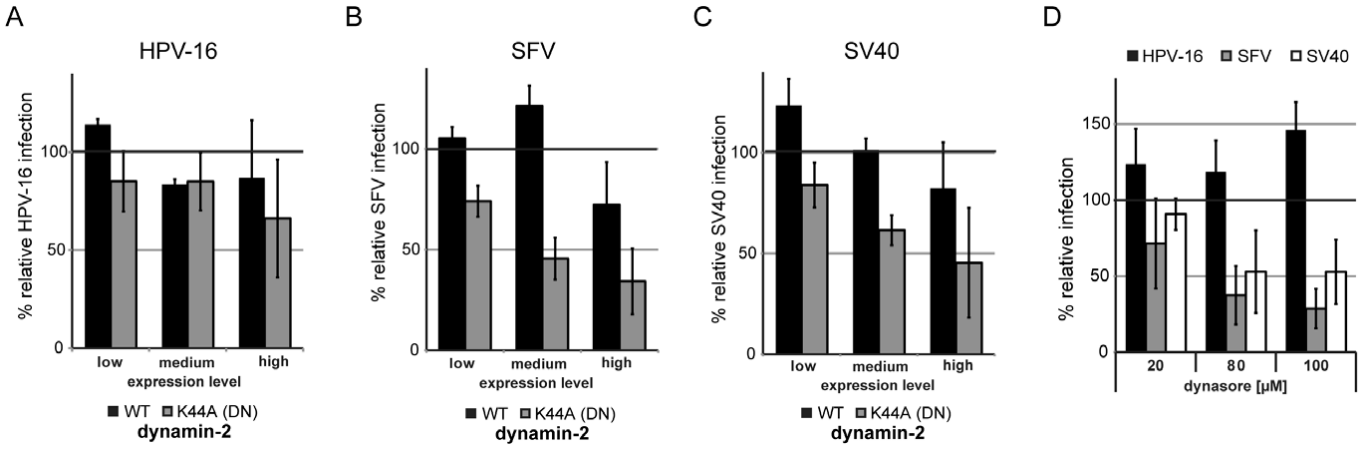
HPV-16 entry is dynamin-2-independent. HeLa cells were transfected with constructs expressing wildtype (WT, black) or the DN K44A (grey) dynamin-2-GFP. 24 h post transfection cells were infected with HPV-16 PsV dsRed (**A**), SFV (**B**), or SV40 (**C**), infection was scored by flow cytometry as described in Figure S2B in relation to the level of overexpressed dynamin-2 (low, medium high). Depicted are results normalized to GFP expressing control cells. (**D**) HeLa cells were pretreated for 30 min with dynasore in the indicated concentrations. Cells were infected with HPV-16 PsV (black), SFV (grey), or SV40 (white) and analyzed as in Figure 2C.

Dynamin-2 facilitates membrane fission to generate endocytic vesicles in several endocytic pathways [41], [42]. To test its involvement in HPV-16 entry, we used overexpression of the DN K44A mutant, the inhibitor dynasore, and siRNA depletion of dynamin-2 as methods of pertubation. Overexpression of K44A dynamin-2 deficient in GTP hydrolysis reduced infection of SFV or SV40 (Figure 3B, C) in a dose dependent manner to 40% or 50% of control, respectively,whereas HPV-16 entry was only slightly perturbed (Figure 3A). Dynamin-2 depletion had no significant effect on HPV-16 infection (Table S1). Dynasore, a noncompetitive inhibitor of dynamin-2, reduced infection of SFV and SV40 to 30% and 40%, respectively (Figure 3D). In contrast, HPV-16 entry was enhanced by up to 50% relative to control (Figure 3D). Our data suggested that dynamin-2 was not required for HPV-16 endocytosis.

### HPV-16 entry is facilitated by actin polymerization dynamics independently of Rho GTPase signaling and actomyosin contractility

Actin polymerization plays a role in several endocytic pathways. During phagocytosis and macropinocytosis it causes outward protrusion of the plasma membrane. In addition, actin polymerization but not outward protrusion of the plasma membrane is required in caveolar/lipid raft endocytosis, the IL-2, the glycosphingolypid enriched endocytic compartment (GEEC), and the Arf6 pathways [41], [42].

**Figure 4:**
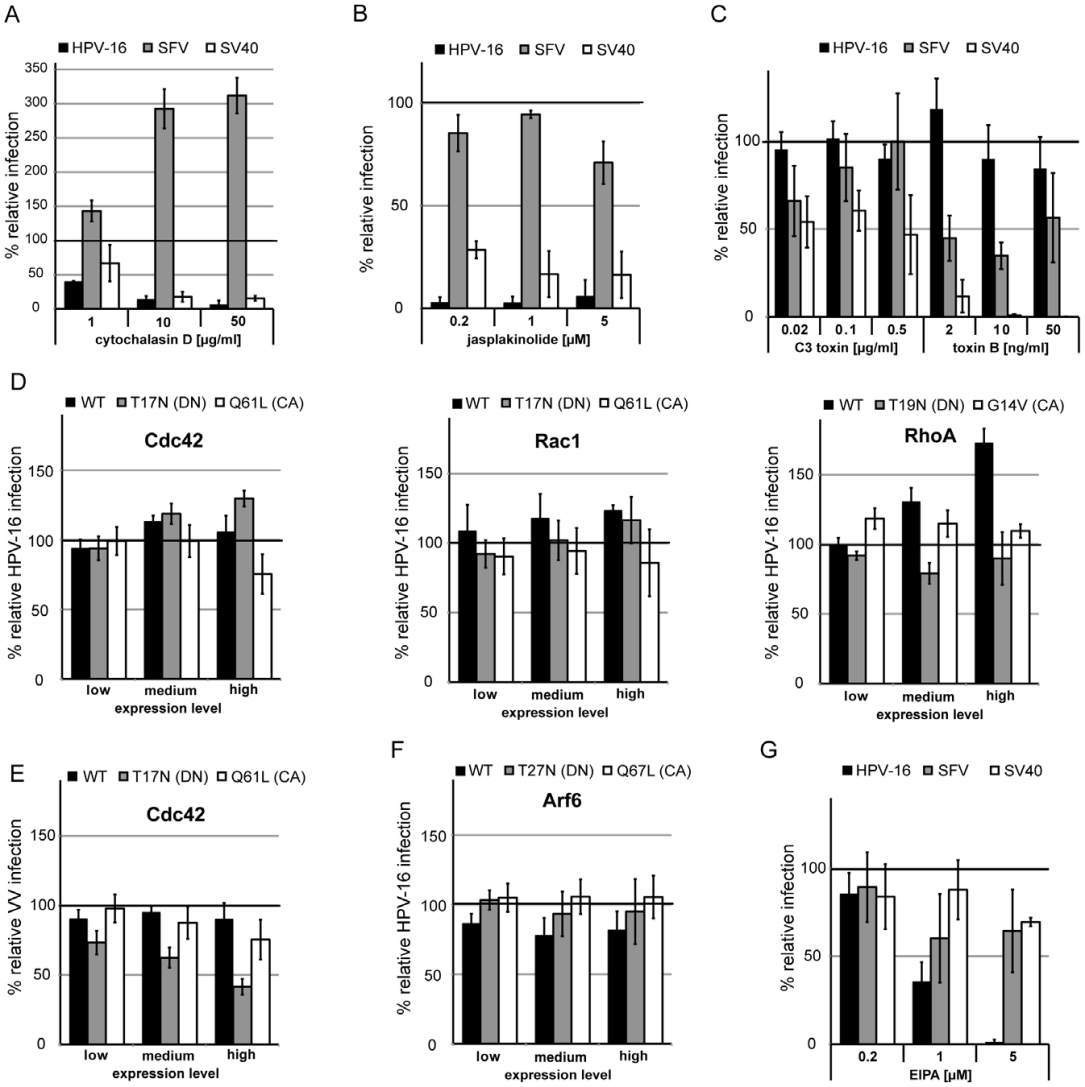
HPV-16 entry requires actin dynamics but not Rho-GTPase or Arf6 signaling. HeLa cells were pretreated for 30 min (A, B, G) or overnight (C) with cytochalasin D (A), jasplakinolide (B), C3 toxin/toxin B (C), and EIPA (G) in the indicated concentrations. Cells were infected with HPV-16 PsV (black), SFV (grey), or SV40 (white) and analyzed as in Figure 2C. Alternatively, HeLa cells were transfected with Cdc42, Rac1, RhoA (D, E), or Arf6 (F) fused to mRFP. Either the wildtype (WT, black), the DN (grey), or the CA (white) mutant were used. 24 h post transfection cells were infected with HPV-16 PsV (D) or VV (E). Infection was scored by flow cytometry as in Figure 3A–C. Depicted are results normalized to mRFP expressing control cells.

To test the involvement of actin, we used the actin depolymerizing agent cytochalasin D and the stabilizing agent jasplakinolide. As expected, the drugs strongly inhibited SV40 infection, whereas SFV infection was unperturbed by jasplakinolide but strongly enhanced by cytochalasin D (Figure 4A, B). Since HPV-16 infection was efficiently inhibited by both drugs (Figure 4A, B, Table S1), we concluded that actin dynamics facilitated infection, in line with previous studies on HPV-16 uptake in BHPE cells or HPV-33 entry into COS-7 cells [23], [43].

Since actin polymerization is most critical during macropinocytic/phagocytic protrusion of the plasma membrane, we checked if HPV-16 infection would be affected by inhibition of the Na^+^/H^+^ exchanger, considered to be a hallmark of macropinocytosis [44]. SFV and SV40 infection were only mildly affected upon EIPA treatment, whereas HPV-16 infection was effectively reduced (Figure 4G), so that we concluded that the Na^+^/H^+^ exchanger is involved.

During macropinocytosis, actin polymerization events are regulated by the Rho-like GTPases Cdc42 and Rac1, whereas phagocytosis and caveolar/lipid-raft endocytosis depend on RhoA [42], [44], [45]. We first inhibited RhoA by C3 toxin, and all Rho-like GTPases by toxin B. C3 toxin, as expected, partially inhibited SV40 infection, but did not affect HPV-16 entry (Figure 4C). Toxin B inhibited SFV infection and SV40 infection, but HPV-16 infection was unperturbed (Figure 4C).

Since Rho-like GTPases are key regulators of actin polymerization, we wanted to verify these results. We overexpressed Cdc42, Rac1, and RhoA, and their DN (T17/19N) or constitutively active (CA, Q61L, G14V) mutants. None of the mutants affected HPV-16 entry (Figure 4D). However, when we infected Cdc42 expressing cells with vaccinia virus (IHD-J/GFP), which enters cells by macropinocytosis [46], [47], the DN mutant blocked infection by about 50% (Figure 4E). Hence, we concluded that HPV-16 infection occurred by an endocytic mechanism dependent on actin polymerization but independent of signaling via the classical Rho-like GTPases.

Arf6, another small GTPase, has been implicated in macropinocytosis and in an elusive endocytic pathway that occurs in HeLa cells [48], [49]. Arf6 also has the potential to stimulate actin polymerization [50]. Hence, we tested whether Arf6 might be regulating actin dynamics during HPV-16 endocytosis. HPV-16 entry was unaffected by overexpression of the CA (Q67L) or DN (T27N) mutant (Figure 4F). In addition, siRNA mediated silencing of Arf6 did not affect HPV-16 entry (Table S1). We concluded that HPV-16 endocytosis did not involve an Arf6 mediated pathway, and that actin dynamics during HPV-16 entry were not regulated by Arf6.

Also, actomyosin contractility was not required for entry, as inhibitors of myosin II and myosin light chain kinase only mildly reduced infection consistent with their role in transport of HPV-16 along filopodia by actin retrograde flow (Table S1, [7]).

### Signaling involved in HPV-16 infection

**Figure 5:**
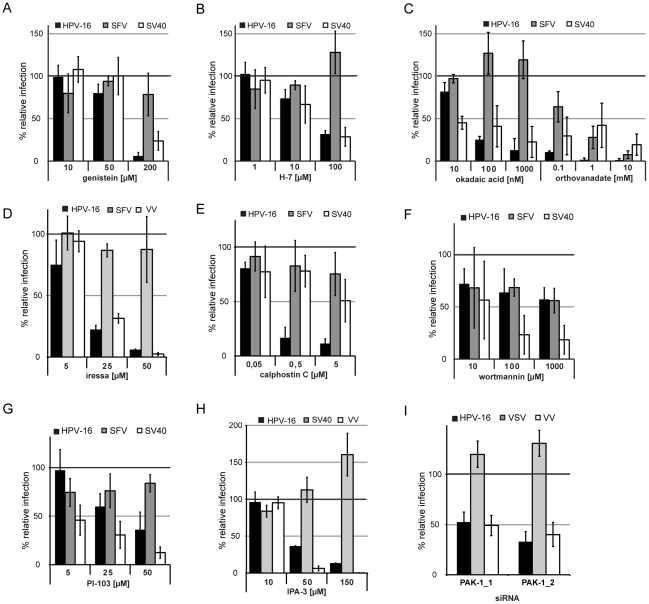
Signaling events required during HPV-16 entry. (A–H) HeLa cells were pretreated for 30 min with genistein (A), H-7 (B), okadaic acid or orthovanadate (C), iressa (D), calphostin C (E), wortmannin (F), PI-103 (G), or IPA-3 (H) in the indicated concentrations. (A–C, E–G) Cells were infected with HPV-16 PsV (black), SFV (grey), or SV40 (white) and analyzed as in Figure 2C. (D) Cells were infected with HPV-16 PsV (black), SFV (grey), or VV (white). Infection was scored by automated microscopy and image analysis as in Figure 2C (HPV-16, VV) or by flow cytometry (SFV). (H) Cells were infected with HPV-16 PsV (black), SV40 (grey) or VV (white). Infection was scored by automated microscopy and image analysis as in Figure 2C (HPV-16, VV) or by flow cytometry (SV40). (I) HeLa cells were transfected with siRNA oligos directed against PAK-1 or with the AllStar Negative control (Qiagen). 48 h after siRNA transfection cells were infected with HPV-16 PsV (black), VSV (grey), and VV (white). Infection was scored as in Figure 2C and depicted relative to cells transfected with the AllStar negative control.

Specific cellular signals are required for activating different endocytic pathways [41], [42], [51]. Hence, we analyzed the role of major kinases and phosphatases involved in endocytic uptake and their effect on HPV-16 entry. Genistein-mediated inhibition of tyrosine kinases blocked HPV-16 entry (Figure 5A). As expected, SV40 infection was likewise affected (Figure 5A, [34], [36]), while SFV infection was unaffected (Fig. 5A). Similarly, inhibition of serine/threonine kinases with H-7 blocked HPV-16 and SV40 infection but not SFV infection (Figure 5B). When we inhibited phosphatases by the broad inhibitor orthovanadate, entry of HPV-16, SV40 and SFV were inhibited (Figure 5C). However, when the phosphatase classes PP1, PP2A, and PP2B were inhibited by okadaic acid, or PP1 by tautomycetin, SFV infection was largely unaffected whereas HPV-16 and SV40 infection was blocked (Figure 5C, Table S1).

To further analyze signaling in the context of HPV-16 endocytosis, we inhibited major kinases involved in macropinocytosis such as EGF receptor tyrosine kinase (EGFR), protein kinase C (PKC), PI3 kinase (PI3K), and p21-activated kinase (PAK-1) [44]. Inhibition of EGFR signaling by Iressa blocked HPV-16 and VV but not SFV entry (Figure 5D). Also, PKC signaling was required for HPV-16 infection as indicated by the inhibitory effects of calphostin C and rottlerin (Figure 5E, Table S1). Inhibition of PI3K by wortmannin or PI-103 reduced HPV-16 infection by 50–60%, which indicated some involvement (Figure 5F, G). Inhibition of PAK-1 by IPA-3 blocked HPV-16 and VV but not SV40 infection (Figure 5H). Also, HPV-16 and VV but not VSV entry were reduced in PAK-1 depleted cells (Figure 5I).

In summary, HPV-16 infection required activation of cellular signaling. The signaling factors included EGFR, phosphatases, PKC and PAK-1, possibly additional tyrosine and serine/threonine kinases, and potentially PI3K. This list of signaling components is similar to the signaling activating macropinocytosis.

To test whether the required cell factors were also critical in more physiologically relevant cells, we infected pharmacologically perturbed HaCaT cells, i.e. spontaneously immortalized keratinocytes. There was virtually no difference between HeLa and HaCaT cells suggesting that HPV-16 entry occurred by a similar endocytic mechanism (Figure S4).

### Cell binding and endocytosis of HPV-16

**Figure 6:**
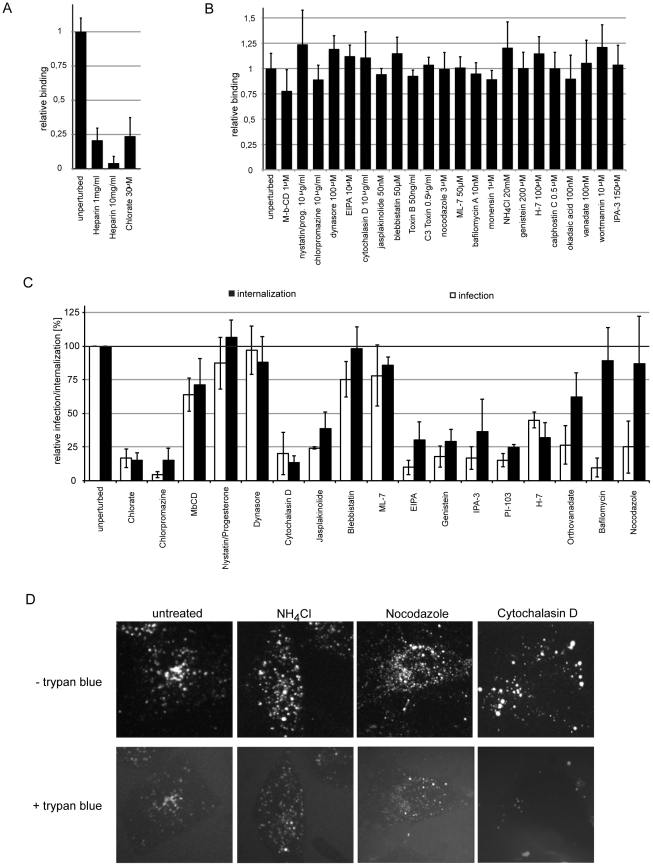
HPV-16 cell binding and internalization. (A, B) HeLa cells were seeded in optical bottom 96-well plates to result in a confluent monolayer at the day of experimentation. Cells were pretreated with chlorate (A, overnight) or further small compound inhibitors (B, 30 min) in the indicated concentrations. Subsequently, HPV-16 AF488 (A, B) or HPV-16 AF488 preincubated with the indicated concentrations of heparin (A) was bound to cells for 1 h, after which cells were fixed with paraformaldehyde. Binding of HPV-16 to cells was analyzed by determining cell associated fluorescence in a sensitive plate reader. The cell associated fluorescence is depicted relative to solvent treated control cells. (C) HeLa cells were pretreated with small compound inhibitors (concentrations as in A, B). Subsequently, cells were infected with HPV-16 PsV in the presence of inhibitor. Cells were treated either treated with high pH buffer at 12 h p.i. to inactivate extracellular virus and infection was continued in the absence of inhbitor (infectious internalization) or the inhibitor was washed out and replaced by NH_4_Cl. (infection). Note that orthovanadate, bafilomycin, and nocodazole inhibit infection but not internalization. The amount of GFP expressing cells relative to solvent treated control cells is given as relative internalization in %. (D) HeLa cells were pretreated with small compound inhibitors for 30 min (concentrations as in A, B). Subsequently, cells were infected with HPV-16 AF594 in the presence of inhibitor. At 6 h p.i., trypan blue (0.008%, w/v) was infused to quench the fluorescence of extracellular virus. Images depict the same cell prior and after trypan blue infusion.

To test whether any of the inhibitors affected HPV-16 binding to cells we measured the association of fluorescent viruses with perturbed HeLa cells. As a positive control, we blocked cell binding by preincubation of PsV with soluble heparin, as previously described [11], [13], [52]. Alternatively, chlorate treatment of cells was used, which results in undersulfation of GAG chains that the virus uses as attachment factors. In both cases, a dramatic reduction in binding was observed (Figure 6A). When we used a variety of inhibitors that did or did not perturb HPV-16 entry, no significant reduction in binding was observed (Figure 6B). Only cholesterol depletion of the plasma membrane by methyl-β-cyclodextrin showed a minor reduction in binding that correlated with a small reduction in infection, which suggested a general membrane-receptor perturbing effect of the drug as nystatin/progesterone had no effect.

To analyze which factors contributed to endocytic internalization as opposed to intracellular events prior to acid-activation we employed the internalization assay for infective particles and compared it to the infection assay with inhibitor swap (compare Figure 1A, Figure S1, S2, material and methods). The relative amount of internalized infectious particles was analyzed at 12 h, when the drug was washed out, and a high pH wash inactivated extracellular virus, so that infection could only be caused by already internalized virus. Infection was continued the absence of an inhibitor for further 36 h. To control for the general reversibility of drug treatment and for cytotoxicity, we included samples where we did not subject cells to a high pH wash. Any samples where reversibility of the inhibitors of interest was significantly affected were excluded. As a positive control, we used chlorate treatment.

Infectious internalization and infection were found to be similarly reduced by cytochalasin D (actin polymerization), jasplakinolide (actin depolymerization), EIPA (Na^+^/H^+^ exchanger), genistein (tyrosine kinases), PI-103 (PI3K), IPA-3 (PAK-1), and H-7 (serine/threonine kinases) (Figure 6C). Although infection was reduced, internalization was only slightly perturbed by treatment of cells with orthovanadate (phosphatases), or unaffected by bafilomycin (endosomal pH), and nocodazole (microtubules) suggesting a role in intracellular routing of the virus. Nystatin/progesteron (cholesterol), blebbistatin (myosin II), and ML-7 (myosin light chain kinase) did not affect HPV-16 internalization or infection, as expected. We concluded that endocytic internalization of HPV-16 required actin polymerization dynamics, the function of the Na^+^/H^+^ exchanger, tyrosine and serine/threonine kinases, PAK-1, and PI3K signaling. Intracellular steps required phosphatases, microtubules, and acid-activation.

Then, we assessed internalization of HPV-16 particles (as opposed to infectious units) using a trypan blue based assay [35]. Addition of the membrane impermeable trypan blue to cells quenched the fluorescence of AF594 labeled particles bound to cells, whereas the fluorescence of internalized particles was unaffected (Figure S5). Microscopic analysis showed that e.g. cytochalasin D rendered almost all HPV-16 particles susceptible to trypan blue consistent with a block in internalization, whereas e.g. NH_4_Cl and nocodazole did not (Figure 6D). Since this data correlated with our infectious internalization analysis (Figure 6C, D; data not shown), we concluded that HPV-16 particles were not internalized and not channeled into a non-infectious uptake pathway but stalled at the cell surface.

### Morphology of HPV-16 internalization

**Figure 7:**
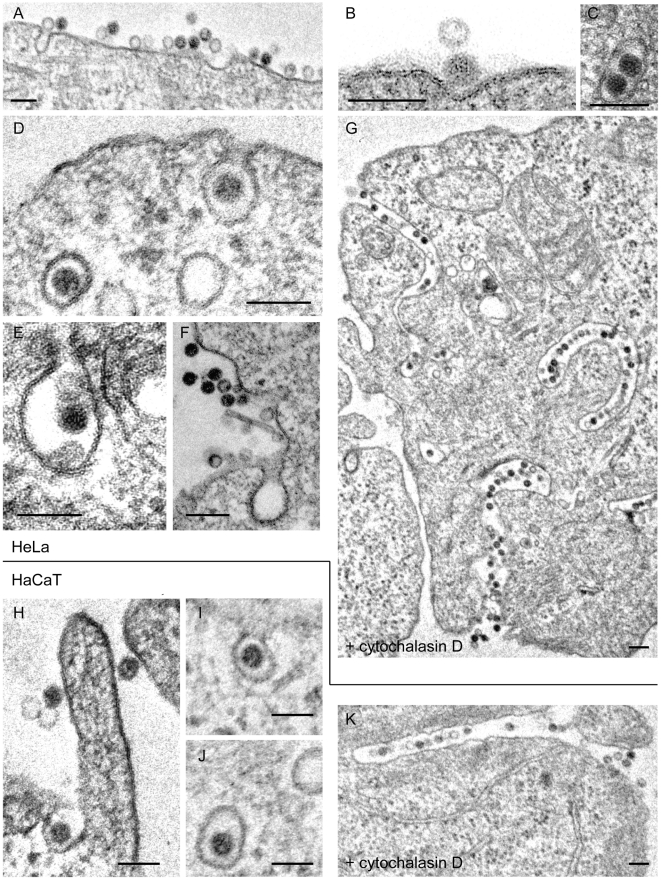
Ultrastructural morphology of HPV-16 internalization. HeLa (A–G) or HaCaT (H–K) cells were infected with HPV-16 PsV (10–20,000 particles/cell) for different times prior to fixation by glutaraldehyde. Cells were either untreated (A–F; H–J) or treated with cytochalasin D (10 µg/ml) throughout infection (G, K). Cells were processed for thin section electron microscopy according to standard procedures. Depicted are representative micrographs for 2–4 h p.i. (A–F; H–J) or 6 h p.i. (G, K). Scale bars represent 100 nm.

Using thin section electron microscopy, we analyzed the morphology of HPV-16 internalization in HeLa and HaCaT cells. Bound viruses were readily detectable at the plasma membrane (Figure 7A). Occasionally, viruses appeared to cause a slight indentation in the plasma membrane (Figure 7B). From 1–24 h post warming, viruses were mainly observed in indentations 65–120 nm in diameter without a visible coat (Figure 7D, H, I) but not in clathrin coated pits (Figure 7F, data not shown). Occasionally, these indentations were wider or tubular and held several particles (Figure 7E, not shown). The particles had a distance of about 5–10 nm from the membrane. About 10–20% were associated with membrane protrusions resembling filopodia, which we have previously shown to be capable of binding and transporting HPV-16 (Figure 7H, [7]). Virus containing indentations were occasionally located at the base of such protrusions (Figure 7H). In close proximity to the plasma membrane, viruses were observed in small vesicles (70–140 nm in diameter) that held one or sometimes two particles (Figure 7C, D, J). Our data suggested that viruses were internalized via such small uncoated pits and vesicles that could accommodate one or possibly several particles.

When virus internalization was blocked using cytochalasin D to inhibit actin polymerization, virus particles were found in deep, narrow, almost tubular invaginations of the plasma membrane that varied in length from a few 100 nm to microns where viruses occurred in tight rows (Figure 7G, K). Thus, the endocytic pits containing viruses seemed to grow into long tubules unable to separate from the plasma membrane by scission. In addition to implicating actin polymerization in scission, this suggested that viruses were internalized after sinking into plasma membrane rather than by protrusive engulfment, which would often be seen during macropinocytosis.

### Intracellular trafficking of HPV-16

After endocytic internalization, most incoming animal viruses are delivered to endosomes and their penetration into the cytosol occurs either in early or late endosomes (EE, LE). If the viruses are endocytosed by macropinocytosis or related mechanisms, the agenda is less well characterized. Macropinosomes can recycle to the plasma membrane or fuse with endosomes or lysosomes at different levels of the pathway [44]. They acidify and it is possible that they undergo a maturation of their own before fusing with lysosomes.

Previous studies have shown that entry of a variety of papillomaviruses is blocked by agents that raise the pH in endosomes and lysosomes [5], [6], [8], [22], [53]. We confirmed that HPV-16 infection, both in HeLa and HaCaT cells, is reduced by the weak base NH_4_Cl, which neutralizes endosomal pH, and by other inhibitors of endosomal acidification such as bafilomycin A1 and monensin (Table S1, not shown). Hence, endocytosis carries viruses to acidic compartments, where low pH-based activation for penetration or uncoating likely occurs.

**Figure 8:**
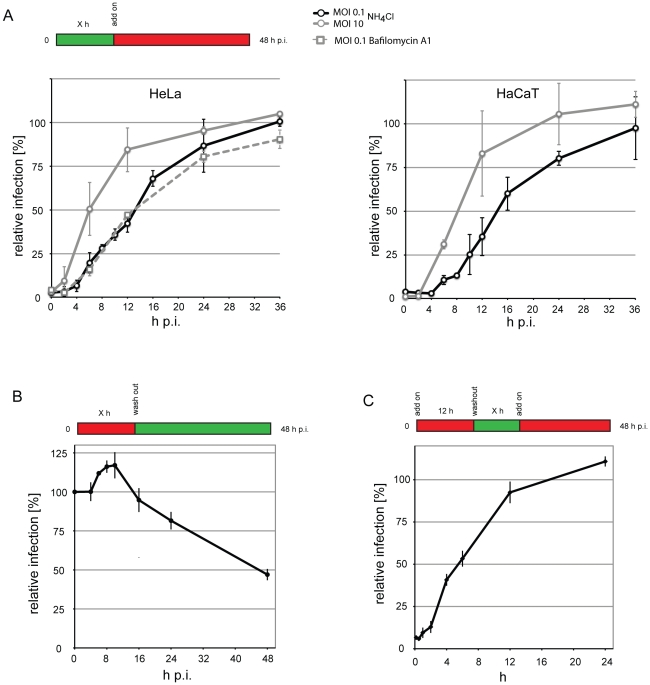
Kinetics of HPV-16 acid-activation during entry. (A) HeLa or HaCaT cells were infected with HPV-16 with different multiplicities of infection (MOI, in transducing units/cell). At different times p.i. NH_4_Cl (straight lines) or Bafilomycin A1 (striated line) was added, and infection was continued in the presence of the drug. (B) HeLa were infected with HPV-16 at MOI 0.1 (10 particles/cell) in the presence of NH_4_Cl (straight line). At different times p.i., the drug was washed out as indicated, and infection was continued in the absence of the drugs. (C) HeLa were infected with HPV-16 as in (B) in the presence of NH_4_Cl. 8 h p.i., the drug was washed out, and infection was continued in the absence of the drug for indicated times, after which NH_4_Cl was re-added and infection was continued in the presence of the drug, thus creating a time window of drug absence. (A–C) Infection was scored 48 h p.i by flow cytometry. Depicted are results normalized to untreated control cells.

**Figure 9:**
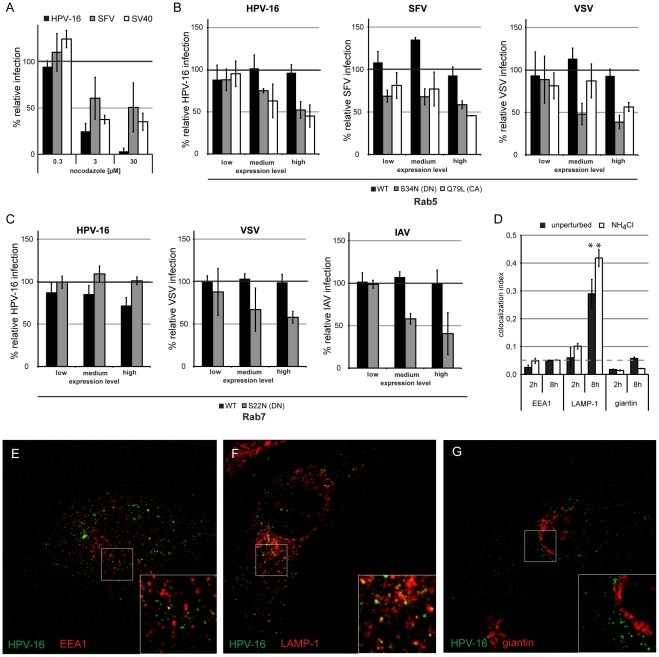
Intracellular vesicular transport of HPV-16 leads virus particles to the late endosomal/lysosomal compartments. (A) HeLa cells were pretreated for 30 min with nocodazole in the indicated concentrations. Cells were infected with HPV-16 PsV (black), SFV (grey), or SV40 (white) and analyzed as in Figure 2C. (B, C) HeLa cells were transfected with Rab5 (B) or Rab7 (C) fused to a mRFP tag. Either the wildtype (WT, black), the DN (grey), or the CA (white) mutant were used. 24 h post transfection, cells were infected with HPV-16 PsV, SFV, VSV, or IAV as indicated. Infection was scored by flow cytometry as in Figure 3A–C. Depicted are results normalized to mRFP expressing control cells. (D) HeLa cells were infected with HPV-16 AF488 for 2 or 8 h, in the presence (white) or absence (black) of 20 mM NH_4_Cl, fixed with paraformaldehyde and immunostained for EEA1, LAMP-1, or giantin. Samples were analyzed by confocal laser scanning microscopy (cLSM) and the relative amount of the colocalization index was determined as described in material and methods. Asterisk indicates a significant Costes P-value. (E–G) Merge images of a single cLSM slice of EEA1 (E), LAMP-1 (F), or giantin (G) (red) and HPV-16 AF488 (green) fluorescence of HeLa cells infected for 8 h.

To determine when the virus passed the acid-activated step during entry, we added NH_4_Cl and bafilomycin A1 at various times post warming and scored for infection. If infection would no longer be perturbed at a certain time, the viruses must have passed the acid-activation step. Our results indicated that about half of the virus population had passed the low pH dependent step by 13–16 h in HeLa and HaCaT cells (Figure 8A). This means that the critical acid exposure occurred 2–3 h after endocytosis (compare Figure 1A, B). We confirmed previous findings that infection can be blocked by nocodazole-induced depolymerization of microtubules suggesting a need for intracellular transport of endocytic vacuoles or the virus itself (Figure 9A, [6], [23]). Since nocodazole add-on experiments followed a similar time course (not shown), we assume that microtubule-based intracellular sorting of endosomes is required for successful HPV-16 infection.

Progression in the endocytic pathway involves the risk that viral particles are degraded. We infected cells at a low MOI in the presence of NH_4_Cl and subsequently washed out NH_4_Cl at various times. The virus remained infectious for prolonged times exhibiting 80% infectivity even after a washout at 24 h p.i. (Figure 8B). Thus, the viruses were not rapidly routed to compartments, where they were inactivated after acidification.

To analyze the average time that viruses require for acid-activation, we infected cells in the presence of NH_4_Cl. After 12 h, we washed out NH_4_Cl which results in a fast re-acidification of endosomes and lysosomes [54]. At various times after washout, we re-added NH_4_Cl, and thus created a time window of virus exposure to low pH. When infectivity was quantified, we found that the halftime of activation was 6 h (Figure 8C), a surprisingly long time period suggesting that to be infectious the virus had to be exposed to low pH, or an enzyme requiring low pH for several hours. That the long time period was specific to HPV-16, is shown by the fact that the low pH based activation of VSV occurred almost completely within a time window of 15 min. (Figure S6).

Since most transport steps in the endocytic pathway are regulated by Rab GTPases, we analyzed which of several RabGTPases were required for HPV-16 infection. We overexpressed GFP- or RFP-tagged Rab GTPases either as wildtype, or in the form of DN or CA mutants.

The DN and CA mutants of Rab5 reduced HPV-16 infection when highly overexpressed (Figure 9B). To control for the efficiency of the dominant effect, we used SFV and VSV as controls, which both require Rab5-mediated transport to EEs for infection. Since the infectivity of both was similarly reduced (Figure 9B), we concluded that HPV-16 required Rab5. No requirement for Rab7 was detected (Figure 9C, left panel). As a positive control, we infected cells with IAV that requires Rab7 for transport to the LE compartment [55]. Since infection of IAV was reduced by the DN mutant, we concluded that HPV-16 was Rab7-independent (Figure 9C, right panel). Overexpression of Rab1, Rab4, Rab6, Rab11 and their mutants did not affect infection (Figure S7).

When the localization of HPV-16 with EE markers was analyzed, we found no significant colocalization of virus with the EEA1 from 30 min -2 h or at 8 h (Figure 9D, E, data not shown). However, we readily found AF488 labeled particles that co-migrated with Rab5 positive structures in live cells (Video S2). The time period spent in the Rab5-positive compartments may be fairly limited as exemplified in Video S2 by a virus entering a Rab5-positive, virus containing vacuole that loses the Rab5 signal about 90 s after this event.

Colocalization of HPV-16 with the LE and lysosome marker LAMP-1 was detectable at 8 h p.i. (Figure 9D, F). Since the localization of LAMP-1 in the crowded perinuclear space may introduce artifactual values for colocalization, we analyzed colocalization with giantin, a Golgi marker. No significant colocalization was observed (Figure 9D, G). We concluded that PsV were trafficked to perinuclear, LAMP-1 containing, acidic compartments. Virus was transported to these vacuoles in the presence of NH_4_Cl (Figure 9D, G). Combined with the fact that after NH_4_Cl washout the virus remained infectious (compare Figure 8B), this result suggested that the transport occurred to LEs or endolysosomes, and that this represented the infectious route.

**Figure 10:**
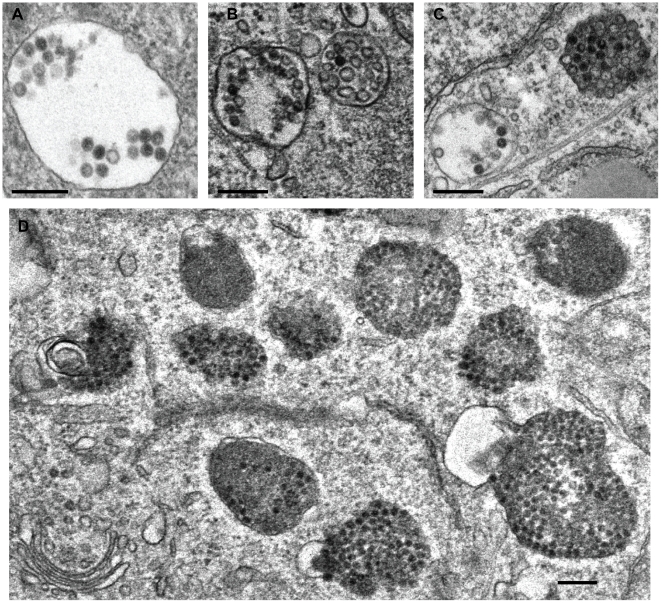
Ultrastructural morphology of HPV-16 intracellular trafficking. HeLa cells were infected with HPV-16 PsV (10–20,000 particles/cell) for 12 h (A, B) or 18 h (C, D) prior to fixation by glutaraldehyde. Cells were processed for thin section electron microscopy according to standard procedures. Scale bars represent 250 nm.

EM showed the virus frequently in multivesicular bodies (Figure 10B, C) and lysosomes (Figure 10D), and occasionally in large endosomal vacuoles that lacked internal vesicles (Figure 10A). They were not observed in the Golgi complex, the endoplasmic reticulum (ER), or the cytosol. Interestingly, virus particles were detectable even late during infection in lysosomal compartments, which suggested that despite the fact that BrdU labeled DNA of PsV become accessible to antibody staining [56], they were not degraded and remained as recognizable particles.

In summary, our data were consistent with intracellular transport of HPV-16 to the LE/lysosomes. During transport the virus-containing vacuoles underwent a transient Rab5 association.

## Discussion

The cell biological experiments presented here indicated that entry of HPV-16 into tissue culture cells involves strategies different from those described for most other viruses. Instead it involves signaling-dependent formation of small invaginations and vesicles at the plasma membrane devoid of detectable coats. The cellular factors needed to generate the PEVs are similar but not identical to those involved in macropinocytosis. After endocytosis, the viruses are deposited into acidic organelles with similarities to classical endosomal compartments and macropinosomes. Clearly, the uptake process in HeLa and HaCaT cells does not depend on CME as previously proposed [5], [6], [8], [22].

Internalization and intracellular trafficking of HPV-16 occurs exceptionally slowly and asynchronously. In agreement with previous reports [6], [23], [24], infectious internalization of a pre-bound cohort of viruses lasted over a period of no fewer than 20 h with a t_1/2_ of 11 h. Judging by the timing of the critical acid-exposure step, a microtubule-dependent step, and the arrival in LAMP-1-containing vacuoles, the average time that viruses spent in endocytic organelles before penetration into the cytosol was 2-3 h. For efficient infection, the viruses had to be exposed to acidic pH in the endosomal pathway for several hours. In comparison, viruses such as VSV and SFV are internalized within minutes and acid-activated penetration takes place a few minutes thereafter (compare Figure S6) [37], [57]. Failing to penetrate, they follow the classical endosome pathway and begin to be degraded in lysosomes within 30 min. The only other viruses with comparatively slow entry kinetics are the polyomaviruses. For example SV40 takes 6–12 h to reach the ER where penetration occurs [58], [59].

Initial binding of HPV-16 particles to the cell surface occurs rapidly and with high affinity [60]. In a mouse vaginal model, binding occurs to the basement membrane and depends on HSPGs [14], [20], whereas in human tissue culture cells ECM binding can in addition involve laminin-332 (previously named laminin-5, [15]). Proteoglycan-bound viruses undergo a structural change that subsequently allows further changes conferred by cyclophilins and furin [16], [17]. A conserved epitope in the minor capsid protein L2 is exposed [18]. This is crucial for infection, and probably followed by transfer of the virus to an unidentified secondary cell surface receptor [18], [21].

Live cell microscopy showed that the internalization event as such is rapid. It occurs on average within 2 minutes once lateral movement of the virus has stopped. It is possible that viruses can only trigger infectious internalization once they are fully activated and have been transferred to the new receptor. This means that the changes induced by cyclophilin and furin, or the transfer to the secondary receptor may be rate-limiting for internalization. This step-wise process would thus occur asynchronously followed by a fast endocytic internalization. Viruses would then be endocytosed one at a time causing a protracted internalization time course seen in the virus population. Since the structural changes occur when the viruses are bound to the ECM as shown in mouse vaginal tissue [20], transfer from the ECM to the cell body may also take a long time. The transfer from ECM to cells is probably promoted by actin- and myosin II-mediated transport of viruses along filopodial cell surface protrusions as observed in tissue culture cells [7].

Since internalization was strongly dependent on cell signaling, it was most likely a virus-induced process. Inhibitor analysis showed that the signaling cascade included the EGFR as well as other tyrosine and serine/threonine kinases such as PAK-1 and PKC. In addition, PI3K signaling was required, which was previously identified to be required for entry of HPV 6b, 18, 31, and 35 or for uptake of HPV-16 into langerhans cells [61], [62]. The involvement of the EGFR and the other downstream signaling kinases was reminiscent of macropinocytosis, which is often activated by receptor tyrosine kinases and requires a series of further kinases. Another kinase that may additionally be involved in HPV-16 endocytosis is the FAK [63]. That the signaling cascade was modulated, was suggested by the requirement for cellular phosphatases.

Endocytosis and infection were clearly independent of CME. Clathrin, AP-2, and dynamin were not needed, and HPV-16 particles did not co-internalize with clathrin-light chain-RFP, or co-localize with endogenous clathrin heavy chain (not shown). Internalization took place in smooth, uncoated pits. That chlorpromazine blocked infection and internalization of HPV-16 particles may be explained by pleiotrophic effects of the drug. Namely, chlorpromazine can interfere with the formation of phagosomes or macropinosomes [64], [65], cause inhibition of PLC-regulated actin rearrangements [66], [67], and change plasma membrane fluidity [68]. Also, HPV-16 entry did not occur by a dynamin-, caveolin-, flotillin-, lipid raft-, or Arf6-mediated mechanism, thus excluding a role for other often reported micropinocytic pathways (compare [41], [51]). Two previous studies implicated caveolin-1 and dynamin-2 in HPV-16 infection in HaCaT cells [4], [69]. However, the observed effects of dynamin pertubation were marginal, cell toxicity due to caveolin-1 depletion high, and no positive and negative controls were employed [4], [69].

Actin polymerization and depolymerization was critical for HPV-16 endocytosis. That long, virus-filled, tubular invaginations were formed in the plasma membrane of cytochalasin D-treated cells suggested that actin was required for scission of endocytic vesicles and not for the generation of membrane protrusions as observed for macropinocytosis. The lack of protrusions was supported by electron microscopy data, and the lack of ruffling or bleb formation by light microscopy ([7], not shown). Moreover, the dynamics of actin were not regulated by the classical Rho GTPases, Cdc42, Rac1, and RhoA. In contrast to macropinocytosis which occurs via large, irregularly shaped, fluid-filled vacuoles, the HPV-16 was observed in small invaginations in the plasma membrane (65–120 nm in diameter). These lacked any visible coat structure. Scission resulted in the formation of small endocytic vacuoles that held one or occasionally two virus particles.

Obviously, many details in the process of vesicle formation remain unclear. We do not know how membrane curvature beneath the virus is induced, how growing pits are stabilized, how and when constriction and membrane scission occur, and how these events are coordinated in time and space. Numerous cellular proteins are likely to be involved in this process. Tetraspanin-enriched microdomains have been suggested as entry platforms for HPV-16 endocytosis [9], and may serve as scaffolds for the recruitment of cytosolic proteins and as a signaling platform.

After endocytosis, the viruses were briefly associated with Rab5-containing vacuoles, and Rab5 was also required for infection. Whether the Rab5-containing structures were EEs is not clear, because no significant colocalization with another marker of EEs, EEA1, could be observed. The viruses were further transported to LE and lysosomes positive for Rab7 and LAMP1. It is possible that the virus bypassed EEs, and like macropinosomes and phagosomes entered the endocytic pathway at the level of LEs or endolysosomes [70].

Two to three hours after internalization the virus passes through a low pH-dependent step critical for infection. It is unclear at this point, whether it involves structural changes triggered in the virus triggered directly by low pH, a block in endosome trafficking and fusion [71], or the impaired function of endosomal proteases [72].

The combination of low pH-dependent activation and action of endosomal proteases after proper trafficking may result in a major uncoating step. There is evidence the viral genome and the minor capsid protein L2 are exposed in a process most likely triggered by the shedding of at least a part of L1 [56]. Remarkably, even after 18 h within LE/lysosomal compartments virus particles appeared to be intact by EM. Potentially, the virus could fully uncoat only after or during translocation into the cytosol. Penetration through the vacuolar membrane has been suggested to involve exposure of a membrane destabilizing peptide in L2 [73]. Before they can enter the nucleus, the viruses use microtubule-dependent transport mediated by an interaction between L2 and dynein [74].

Taken together, the cell biological features of HPV-16 endocytosis and intracellular trafficking showed several similarities with macropinocytosis. In Table 1, we have listed some of the known properties of macropinocytosis as well as their manifestation during vaccinia virus entry. When compared with the results on HPV-16 endocytosis, the strong dependence of HPV-16 on actin dynamics, receptor tyrosine kinases, PKC, PAK-1, and the Na^+^/H^+^ exchanger are consistent with a macropinocytic mechanism [44]. In particular, the requirement for receptor tyrosine kinases, PAK-1 and the Na^+^/H^+^ exchanger suggest that the signaling pathway is shared [44]. However, HPV-16 endocytosis did not involve formation of ruffles or other types of plasma membrane protrusions. The role of actin was rather connected to the pinching off of endocytic vesicles. Cholesterol-rich domains, myosin II and Rho GTPases were not involved, and the HPV-16 containing endocytic pits and vesicles were small and relatively homogeneous in shape and diameter. Another difference was that endocytosis of HPV-16 occurred over prolonged periods of time and seemed to activate the process locally, whereas e.g. a single vaccinia virus has been shown to activate macropinocytosis transiently for about 30 min and on large areas of the plasma membrane. We have to conclude that HPV-16 endocytosis did not occur by normal macropinocytosis. The data also did not conform with what is known about phagocytosis (Table 1).

**Table 1:** Comparison of the cellular requirements for macropinocytosis, VV, IAV CME-independent, and HPV-16 endocytosis, and phagocytosis.

Macropinocytosis (stimulated by growth factors)	VV	Influenza A Virus	HPV16	Phagocytosis (Fc receptor)	Phagocytosis (CR3)	Characteristics
ruffling	blebbing	n.d.	small pits*	phagocytic cups	PM indentation	PM morphology
✓	✓	✓	n.d.	✗	✗	Fluid uptake
✓	✓	n.d.	✗*	✓/✗	✓/✗	cholesterol
✓	✓	✓	✓	✓	✓	Actin
✓	✓	✓	✗*	✗	✓	Myosin
✓	✓	✓	✓	✓	✓	Tyrosine Kinases
✓	✓	n.d.	✓	n.d.	n.d.	Ser Thr Kinases
✓	✓	✓	✓	✓	✓	PI(3)kinase
✓	✓	✓	✓	✓	✓	Protein kinase C
✓	✓	✓	✓	n.d.	n.d.	PAK-1
✓	✓	✗	✗*	✓	✗	Cdc42
✓	✓	✗	✗*	✓	✗	Rac1
✗	✗	n.d.	✗*	✗	✓	RhoA
✓	✓	✓	✓	✗	✗	Na+/H+ exchanger
✓/✗	✗	✗	✗	✓	✓	Dynamin-2
✗	✗	n.d.	✓	✓	✓	Arf1
✓/✗	✓	n.d.	✗	✓	✓	Arf6
✗	✗	n.d.	n.d.	✓	✓	AP1
✓/✗	✓	✓	✓	✓/✗	✓/✗	Acidification
✓	✓	✓	✓	✓	✓	Rab5
✓	✓	✓	✗	✓	✓	Rab7

Depicted are characteristics and required factors for macropinocytosis, phagocytosis, and endocytic entry of VV, IAV, and HPV-16. Tick marks indicate that a factor is required, crosses indicate no requirement, both indicate a cell type/system dependend requirement, and n.d. = not determined. Marked with an asterisk (*) are differences between macropinocytosis and HPV-16. The data is from this manuscript and (Mercer and Helenius, 2008, Mercer and Helenius 2009, de Vries et al., 2011, Niedergang and Chavrier, 2005).

HPV-16 is most likely not the only virus using this particular endocytic mechanism for entry. Although IAV can use CME for uptake, electron and light microscopy have indicated the presence of a parallel, macropinocytosis-like alternate pathway involving small pits and vesicles devoid of a clathrin coat [75], [76]. Recent analysis of this pathway revealed dependence on a set of cellular factors similar to those used by HPV-16 (Table 1, [77]). Note, that like HPV-16 this pathway does not depend on Rho GTPases. Other viruses that may follow similar pathways include echo virus 1, a picorna virus, and lymphocytic choriomeningitis virus, an arena virus [78], [79]. A more detailed cell biological analysis of these viruses will be needed to define whether the mechanims are the same, whether they too represent variants of macropinocytosis different enough to be considered a distinct endocytosis pathway altogether.

In respect to papilloma viruses, it will be important to determine if the pathway described here applies to HPV-16 infection in epithelial tissue cells *in situ*. The common findings on the role of actin [23], [43] and on the requirement of PI3K [61], [62] in different cell types and with HPV subtypes, together with divergent results on the use of lipid rafts and caveolin for e.g. HPV-31, warrant a comparative study of different HPV subtypes and papillomaviruses from warts and raft cultures to adress whether the majority of HPV subtypes makes use of the same pathway in a variety of cell types.

## Materials and Methods

### Cells, antibodies, small compound inhibitors, siRNAs, plasmids

HeLa and HaCaT cells, and mouse embryonic fibroblasts (caveolin-1 wildtype and knockout, [35]) were cultured in DMEM (Invitrogen) containing 10% fetal calf serum. For a full list of antibodies, inhibitors, plasmids, siRNAs, or reagents used, please refer to Table S2.

### Viruses

HPV-16 PsV containing the pRwB or pCIneo-EGFP plasmid were produced with the p16sheLL plasmid by the propagation method described by Buck and colleagues [80]. The PsV were matured for 24 h in the presence of RNAse to maximize the purification of PsV containing the reporter plasmid, resulting in an improved particle to infectivity ratio [80]. All plasmids and production methods are fully described on the Schiller laboratory's website (http://ccr.cancer.gov/staff/staff.asp?profileid=5637). IAV (H1N1, A/PR 34/8), SFV, SV40, recombinant VSV (Indiana) expressing GFP (VSV-GFP), recombinant VV (IHD-J) expressing GFP were produced as described [32], [47], [57], [59], [81].

### Fluorescent labeling of HPV-16 PsV

HPV-16 PsV were covalently labeled with fluorophores as described [7]. Briefly, purified HPV-16 PsV were incubated for 1 h at room temperature in PBS with a ten-fold molar excess of Alexa Fluor (AF) succinimidylesters (Molecular Probes) over the major capsid protein L1. PsV were separated from the labeling reagent by size exclusion chromatography using NAP5 columns (GE Healthcare) and stored at 4°C.

### Transfection and transient expression

For transfection, cells were trypsinized, pelleted, washed with PBS and transfected with expression plasmids in Nucleofector solution R (Amaxa) utilizing program I13 of the Amaxa Nucleofector according to the manufacturer's instructions. Cells were seeded in 12-well plates or on 18 mm coverslips and used for infection or live cell imaging experiments, respectively, at 6–14 h post transfection.

### TIRF microscopy

Microscopy was performed on a custom modified Olympus IX71 inverted microscope. Modifications included a heated incubation chamber that surrounded the microscope stage set to 37°C, an objective-type TIRFM setup from TILL Photonics (Grafeling, Germany), and a monochromator for epifluorescence excitation with a controller allowing hardware-controlled fast switching between total internal reflection fluorescence and epi-fluorescence excitation and acquisition (TILL Photonics). Images were acquired using a TILL Image QE chargecoupled device camera and TILLVISION software (both from TILL Photonics). The total internal reflection angle was manually adjusted for every experiment. Live HeLa cells on 18-mm coverslips were mounted in custom-made chambers. 5,000 HPV-16 PsV/cell labeled with AF dyes were added into the 0.5 ml of medium on the stage. Movies were recorded at a rate of 2–5 frames per s.

### Live cell confocal microscopy

Live cell confocal microscopy was performed on a Zeiss 510 microscope with a confocal laser scanning setup. The confocal microscope was equipped with a heated incubation chamber set at 37°C. Cells were mounted on 18-mm coverslips in custom-made chambers, and cells were incubated with normal growth medium. HPV-16 PsV (500–1,000 particles/cell) were added into the 0.5 ml of medium on the stage. After 30 min – 4 h post addition of virus particles to cells image acquisition was performed. Movies were recorded at a rate of 2 frames per s.

### Infection studies and analysis by automated microscopy

For siRNA mediated knockdown experiments, 3,000 cells were reverse transfected in optical bottom 96-well plates (Nunc) with the indicated amount of siRNA oligos using Lipofectamine RNAimax (Invitrogen), and infected 48 h post transfection of siRNAs. In case of oligos directed against clathrin heavy chain and the AP2 μ subunit, cells were transfected for a second time 24 h post initial transfection. For small compound inhibition studies, 5,000 cells were seeded 24 h prior to experimentation in 96-well optical bottom well plates. Inhibitors were added at indicated concentrations 30 min or as indicated 12–16 h prior to infection (Figure S2A). Cells were infected with HPV-16, SV40, SFV, or VV to result in about 20% infection. For inhibitor studies, the primary inhibitors were exchanged at 12 or 10 h p.i. against 10 mM NH_4_Cl or 5 mM DTT in case of HPV-16 or SV40 infection, respectively, to reduce cytotoxicity (Figure S2A). After 4% paraformaldehyde fixation, cells were stained with Hoechst 33258 for cell nuclei and in case of SFV, SV40, and IAV with antibodies directed against SFV glycoproteins, T-antigen, and nucleoprotein, respectively, and secondary antibodies conjugated to Alexa Fluor 488 (Molecular Probes) directed against the primary antibodies. Nine images per well were acquired for each, nuclear stain (Hoechst 33258) and infection stain (GFP, or IF staining for SV40 T-antigen, SFV glycoproteins), on a Pathway 855 automated microscopy station (Becton Dickinson) using a 10× objective (Olympus) employing a laser-based autofocus every image. Cell numbers and raw infection indices for each well were determined using a MATLAB-based infection scoring procedure (The Mathworks) described previously ([35], Figure S2C). The raw infection indices were normalized to solvent treated control cells or AllStar negative siRNA transfected control cells to result in relative infection percentages.

### Infection studies by flow cytometry

5×10^4^ cells seeded in 12-well plates were either transfected as described above or treated with small compound inhibitors 30 min prior to infection. Cells were infected with HPV-16 dsRed or GFP, VSV-GFP, VV-GFP, or IAV to result in 20% infection. Cells were trypsinized, fixed in 4% paraformaldehyde, and in case of IAV, immunofluorescence staining for the nucleoprotein was performed. Transfected cells were analyzed by flow cytometry for the level of transgene expression, grouped into low-, medium-, and high-expressing cells (Figure S2C), and the subpopulation was subsequently analyzed for the number of infected cells. The relative number of infected cells was normalized to cells expressing only the XFP-tag according to expression levels. Inhibitor treated cells were normalized to solvent treated control cells.

### Immunofluorescence staining and analysis

5×10^4^ cells were seeded on 18 mm coverslips and infected 16–24 h post seeding with 500 AF488-labeled HPV-16 particles/cell for 2 or 8 h. Cells were fixed with 4% paraformaldehyde or in ice-cold methanol, stained against cellular structures with primary antibodies (Table S2) and AF594 labeled secondary antibodies (Molecular Probes). Cells were mounted in Citifluor AF1, and analyzed on a Zeiss 510 microscope with a confocal laser scanning setup. Per experiment, at least 10 fields of view were imaged in 5 confocal slices with 3–8 cells/field of view. The degree of colocalization between virus and cellular structures was assessed using Bioimage XD (www.bioimagexd.net). The thresholded pixel-per-pixel colocalization was analyzed in an automated fashion where significance of colocalization was attributed to a Costes P-value≥0.95 [82].

### Binding of HPV-16

30,000 Hela cells were seeded into optical bottom 96-well plates 24 h prior to experimentation to reach confluency. Cells were pretreated with inhibitors as in the infection studies. AF488 labeled HPV-16 (5,000 particles/cell) was added to each well, and incubated for 1 h at 37°C, when cells were fixed in paraformaldehyde and stained with Hoechst 33258 for cell nuclei. Cellular and cell associated fluorescence was analyzed using a Safire^2^ plate reader (Tecan), normalized to solvent controls, and depicted as relative cell associated fluorescence (relative binding).

### Internalization of HPV-16 particles

5×10^4^ HeLa cells were seeded on glass coverslips 24 h prior to experimentation. Cells were infected with HPV-16 AF594 (500–1000 particles/cell) in the presence or absence of inhibitors. 6 h post addition, coverslips were mounted in custom made chambers, and the amount of fluorescent viruses/cell was analyzed in live cells on a Zeiss Observer Z1 microscope equipped with a Yokogawa spinning disc confocal unit and a heating chamber surrounding the microscope by acquiring Z stacks. After the initial acquisition, trypan blue (0.4% (w/v), Invitrogen) was added to live cells in a 1∶50 dilution. This immediately shifted the emission spectrum of viruses on the cell-surface and led to a loss of detectable fluorescence, whereas the fluorescence of intracellular viruses remained unaltered. Images were visually analyzed and representative examples were depicted as maximum intensity projections.

### Infectious internalization of HPV-16

5×10^4^ HeLa or HaCaT cells were seeded 24 h prior to experimentation in 12 well plates. HPV-16 PsV were added to cells in the cold. After 4 h the plates were shifted to 37°C. At different time points post warming, cells were incubated with high pH buffer (PBS, pH 10.5) for 1 min, which was replaced by normal growth medium. The treatment did not cause any cell toxicity (not shown) but resulted in disassembly of surface bound virions rendering them non-infectious (Figure S1). Alternatively, HeLa cells were infected in the presence or absence of inhibitors with HPV-16 to result in about 20% infection of the unperturbed control (10–30 particles/cell) At 12 h p.i., cells were incubated for 2 min with the high pH buffer, which was replaced with normal growth medium without inhbitor causing infection by already internalized virus. 48 h p.i., cells were trypsinized, fixed in 4% paraformaldehyde, and analyzed for infection (GFP expression) by flow cytometry.

### Electron microscopy

For thin-section EM, HeLa or HaCaT cells plated onto 12-mm coverslips were incubated with HPV-16 PsV (10–20,000 particles/cell) 37°C for 30 min, 2, 6, 12, and 24 h before fixation with 2.5% glutaraldehyde (in 0.05 M sodium cacodylate pH 7.2, 50 mM KCl, 1.25 mM MgCl_2_, and 1.25 mM CaCl_2_, 30 min at RT), followed by 1.5 h of incubation in 2% OsO_4_ on ice. Dehydration, embedding, and thin sectioning were performed as previously described [58]. After transmission electron microscopy (Zeiss EM 91 microscope), images were exported as 8-bit TIFF files.

If not otherwise mentioned, all data is depicted ± standard deviation from at least three independent experiments.

## Supporting Information

Figure S1 **HPV-16 infectious internalization assay.** (A) HPV-16 PsV were bound to HeLa cells for 4 h in the cold. Cells were submitted to a 1 min wash with buffer at indicated pH, after which cells were washed with medium, and incubated at 37°C for 48 h. Infection was scored by flow cytometry as in Figure 3A–C. Depicted are results normalized to pH 7.4 treated control cells. (B) Electron micrographs of negatively stained HPV-16 PsV incubated in buffers at indicated pHs. Note the complete disassembly of virions at pH 10.5.
(TIF)

Figure S2 **Inhibitor-based infection studies and analysis.** (A) Schematic representation of the cellular inhibitor studies for virus infections. Cells were pretreated with tested drugs for 30 min prior to infection (green), if not mentioned otherwise. If indicated, drugs were re-added 5 h p.i to ensure efficient inhibition. Infection was carried out for indicated times in the presence of the drug (orange), when cells were fixed (blue). For HPV-16 and SV40, tested inhibitor were exchanged for NH_4_Cl and DTT blocking acid-activation or escape from the ER, respectively (red). (B) Scatter plot of HPV-16 infected GFP transfected HeLa cells. The PsV expressed RFP (y-axis) upon successfull infection. Transgene expression levels (x-axis) were grouped into untransfected, low, medium, and high expressing cells. (C) Infection was analyzed by automated microscopy of samples. Given are exemplary pictures of nuclear stain (Hoechst), infection (GFP/AF488), and merges for HPV-16, SV40, and SFV infections. The data was computationally analzyed, the raw infection index (infected cells, i.e. green/cell number, i.e. green + red) was obtained, and data was normalized to solvent treated control cells on the same plate.
(TIF)

Figure S3 **siRNA mediated knockdown of clathrin, AP2, caveolin-1, flotillin-1 and -2.** HeLa cells were transfected with siRNA oligos directed against the AP2μ-subunit (AP2), clathrin heavy chain (CHC) (A), caveolin-1 (B), flotillin-1, flotillin-2 (C), or as control against luciferase (A) or an AllStar negative control (Qiagen, B, C). Protein lysates were subjected to SDS-PAGE and Western Blotting with antibodies directed against CHC, AP2μ, caveolin-1, flotillin-1, flotillin-2, and actin (loading control) as indicated. Signals were quantified and normalized to the loading control and depicted as relative protein abundance (right panel).
(TIF)

Figure S4 **Cellular requirements for HPV-16 infection in HeLa vs. HaCaT cells.** HeLa (black) and HaCaT (white) cells were pretreated with inhibitors at indicated concentrations for 30 min, and subsequently infected with HPV-16 PsV. 12 h p.i. inhibitors were exchanged for NH_4_Cl, and infection was continued for further 36 h. Cells were fixed and analyzed for GFP expression (infection) by flow cytometry, and infection is given relative to solvent treated control cells in %. Depicted are the results for three independent experiments ± SD.
(TIF)

Figure S5 **Trypan blue-based internalization assay of HPV-16 particles.** HeLa cells were infected with AF594 labeled HPV-16 for 1 h (bound virus) or 12 h (internalized virus). Images of live cells are depicted before (−) and after (+) addition of trypan blue that quenches the fluorescence of externally located virus.
(TIF)

Figure S6 **Kinetics if VSV acid activation in HeLa cells.** HeLa were infected with VSV the presence of NH_4_Cl. 30 min p.i., the drug was washed out, and infection was continued in the absence of the drug for indicated times, after which NH_4_Cl was re-added and infection was continued in the presence of the drug, thus creating a time window of drug absence. Infection was scored 6 h p.i by flow cytometry. Depicted are results normalized to untreated control cells.
(TIF)

Figure S7 **HPV-16 entry is Rab1, Rab4, Rab6, and Rab11-independent.** HeLa cells were transfected with Rab1 (A), Rab4 (B), Rab6 (C), or Rab11 (D) fused to a GFP tag. Either the wildtype (WT, black), the DN (grey), or the CA (white) mutant were used. 24 h post transfection cells were infected with HPV-16 PsV expressing dsRed. Infection was scored by flow cytometry as in Figure 3A–C. Depicted are results normalized to GFP expressing control cells.
(TIF)

Table S1 **Additional infection data in the context of cell perturbations.** The upper table summarizes data on virus infectivity after pharmacological inhibition of cellular targets. HeLa cells were pretreated for 30 min with pharmacological inhibitors in the indicated concentrations. Cells were infected with HPV-16 PsV, SFV, or SV40 in the presence of inhibitor as indicated. Inhibitors were exchanged for HPV-16 and SV40 with NH_4_Cl or DTT, respectively. Infection was scored by automated microscopy and image analysis. Depicted are results normalized to solvent treated control cells. See also Figure S2, and material and methods. The lower table summarizes data on virus infectivity after siRNA mediated depletion of cellular targets The efficiency of the knockdown is expressed as the amount of residual mRNA still present after siRNA transfection. HeLa cells were transfected with siRNA oligos directed against the indicated targets. Cells were infected with HPV-16 PsV and infection was scored 36 h p.i. by automated microscopy and image analysis. Depicted are the mean infection percentages relative to the the AllStar negative control.
(PDF)

Table S2 **List of the antibodies, pharmacological inhibitors, plasmids, siRNAs including their suppliers that were used in this study.**
(PDF)

Video S1 **HPV-16 internalization occurs independently of clathrin.** HeLa cells were transfected with CLC-mRFP (red). About 12–16 h post transfection HPV-16 AF488 labeled particles (green) were added to cells, and imaged 30 min post addition by TIRFM. Imaging occured at 2 fps, movie played at 10 Hz. Shown is a cropped area of a single cell. Encircled in white is the area where an HPV-16 particle becomes confined and is internalized around halfway through the movie. The encircled area shows the internalization event that is depicted as kymograph in Figure 1C.
(MOV)

Video S2 **HPV-16 association with Rab5.** HeLa cells were transfected with Rab5-mRFP (red). About 12–16 h post transfectiom HPV-16 AF488 labeled particles (green) were added to cells, and imaged 3.5 h post addition. Imaging occured at 0.5 fps. Shown is first the whole cell, from where an area oft he cell periphery is zoomed in. Encircled in white is the area where an HPV-16 particle started moving towards a Rab5/virus positive structure, with which it associated, and from where it trafficked towards the nucleus. During the latter transport the virus lost association with the Rab5 signal.
(MOV)
