# Supplementary figures and images for: Entry of Human Papillomavirus Type 16 by Actin-Dependent, Clathrin- and Lipid Raft-Independent Endocytosis
Source: PLoS Pathog. 2012 Apr 19;8(4):e1002657. doi: 10.1371/journal.ppat.1002657 (PMC3334892; doi:10.1371/journal.ppat.1002657)

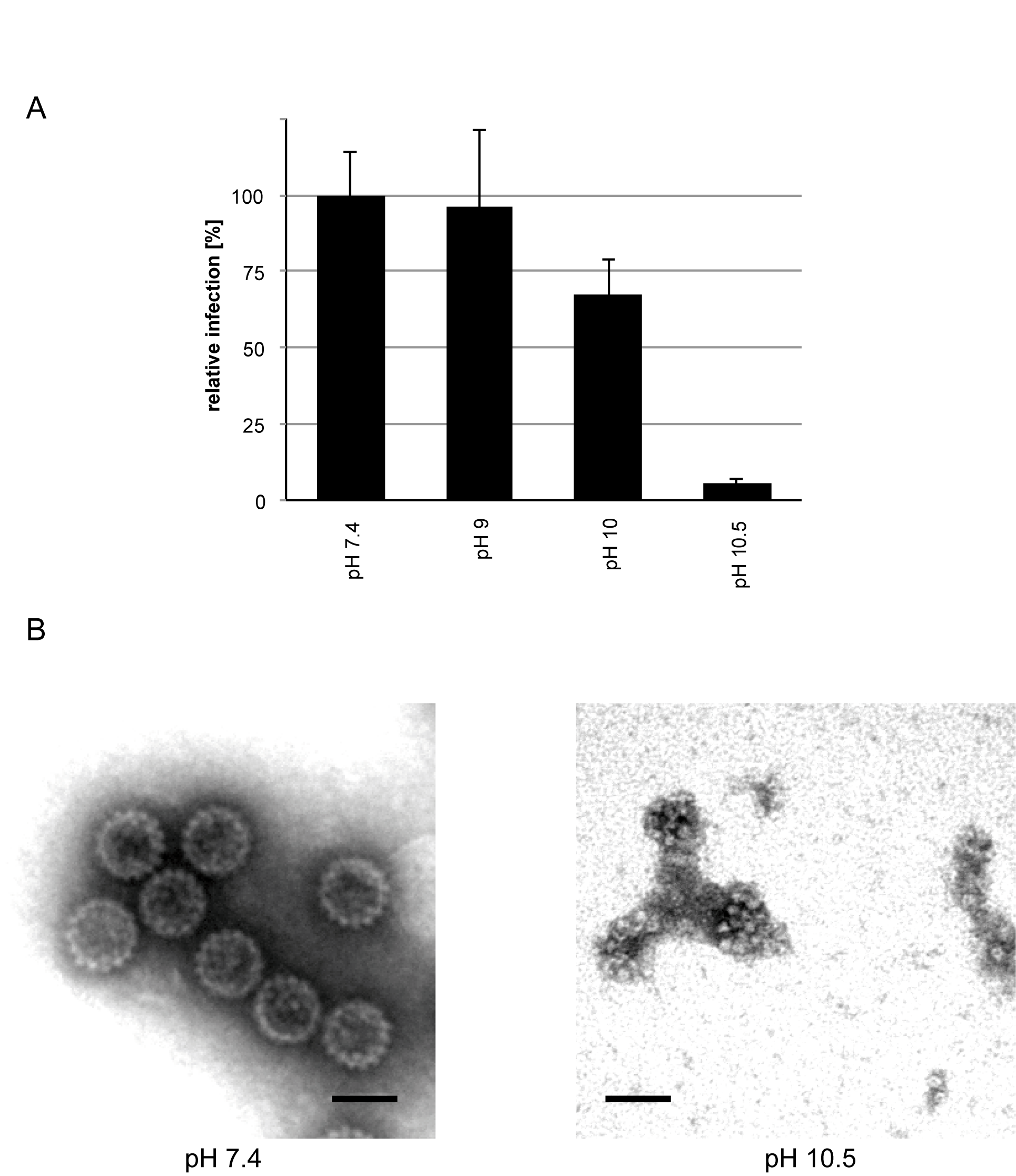

Supplement: Figure S1 — HPV-16 infectious internalization assay. (A) HPV-16 PsV were bound to HeLa cells for 4 h in the cold. Cells were submitted to a 1 min wash with buffer at indicated pH, after which cells were washed with medium, and incubated at 37°C for 48 h. Infection was scored by flow cytometry as in Figure 3A–C. Depicted are results normalized to pH 7.4 treated control cells. (B) Electron micrographs of negatively stained HPV-16 PsV incubated in buffers at indicated pHs. Note the complete disassembly of virions at pH 10.5. (TIF) [file ppat.1002657.s001.tif]

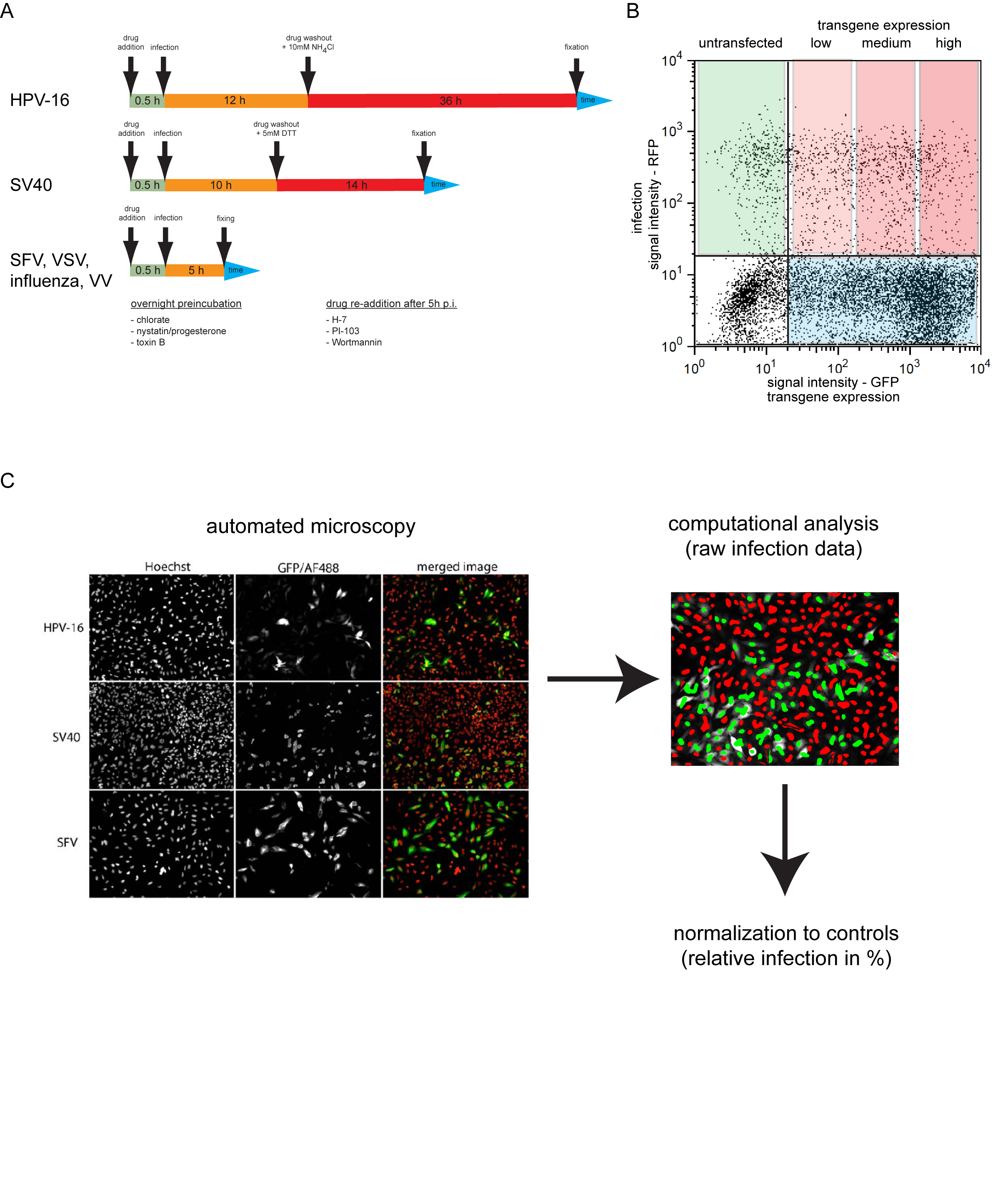

Supplement: Figure S2 — Inhibitor-based infection studies and analysis. (A) Schematic representation of the cellular inhibitor studies for virus infections. Cells were pretreated with tested drugs for 30 min prior to infection (green), if not mentioned otherwise. If indicated, drugs were re-added 5 h p.i to ensure efficient inhibition. Infection was carried out for indicated times in the presence of the drug (orange), when cells were fixed (blue). For HPV-16 and SV40, tested inhibitor were exchanged for NH4Cl and DTT blocking acid-activation or escape from the ER, respectively (red). (B) Scatter plot of HPV-16 infected GFP transfected HeLa cells. The PsV expressed RFP (y-axis) upon successfull infection. Transgene expression levels (x-axis) were grouped into untransfected, low, medium, and high expressing cells. (C) Infection was analyzed by automated microscopy of samples. Given are exemplary pictures of nuclear stain (Hoechst), infection (GFP/AF488), and merges for HPV-16, SV40, and SFV infections. The data was computationally analzyed, the raw infection index (infected cells, i.e. green/cell number, i.e. green + red) was obtained, and data was normalized to solvent treated control cells on the same plate. (TIF) [file ppat.1002657.s002.tif]

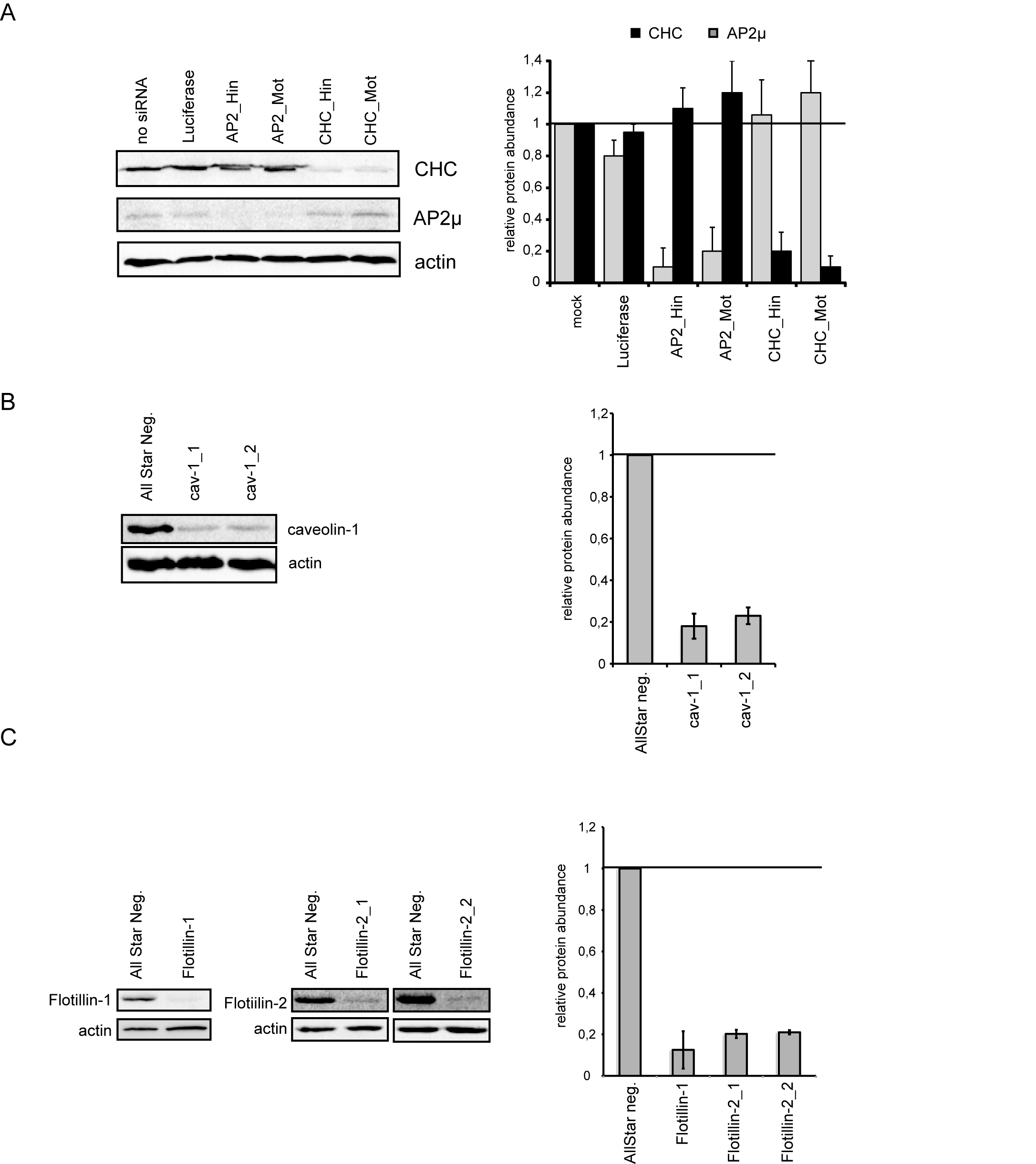

Supplement: Figure S3 — siRNA mediated knockdown of clathrin, AP2, caveolin-1, flotillin-1 and -2. HeLa cells were transfected with siRNA oligos directed against the AP2μ-subunit (AP2), clathrin heavy chain (CHC) (A), caveolin-1 (B), flotillin-1, flotillin-2 (C), or as control against luciferase (A) or an AllStar negative control (Qiagen, B, C). Protein lysates were subjected to SDS-PAGE and Western Blotting with antibodies directed against CHC, AP2μ, caveolin-1, flotillin-1, flotillin-2, and actin (loading control) as indicated. Signals were quantified and normalized to the loading control and depicted as relative protein abundance (right panel). (TIF) [file ppat.1002657.s003.tif]

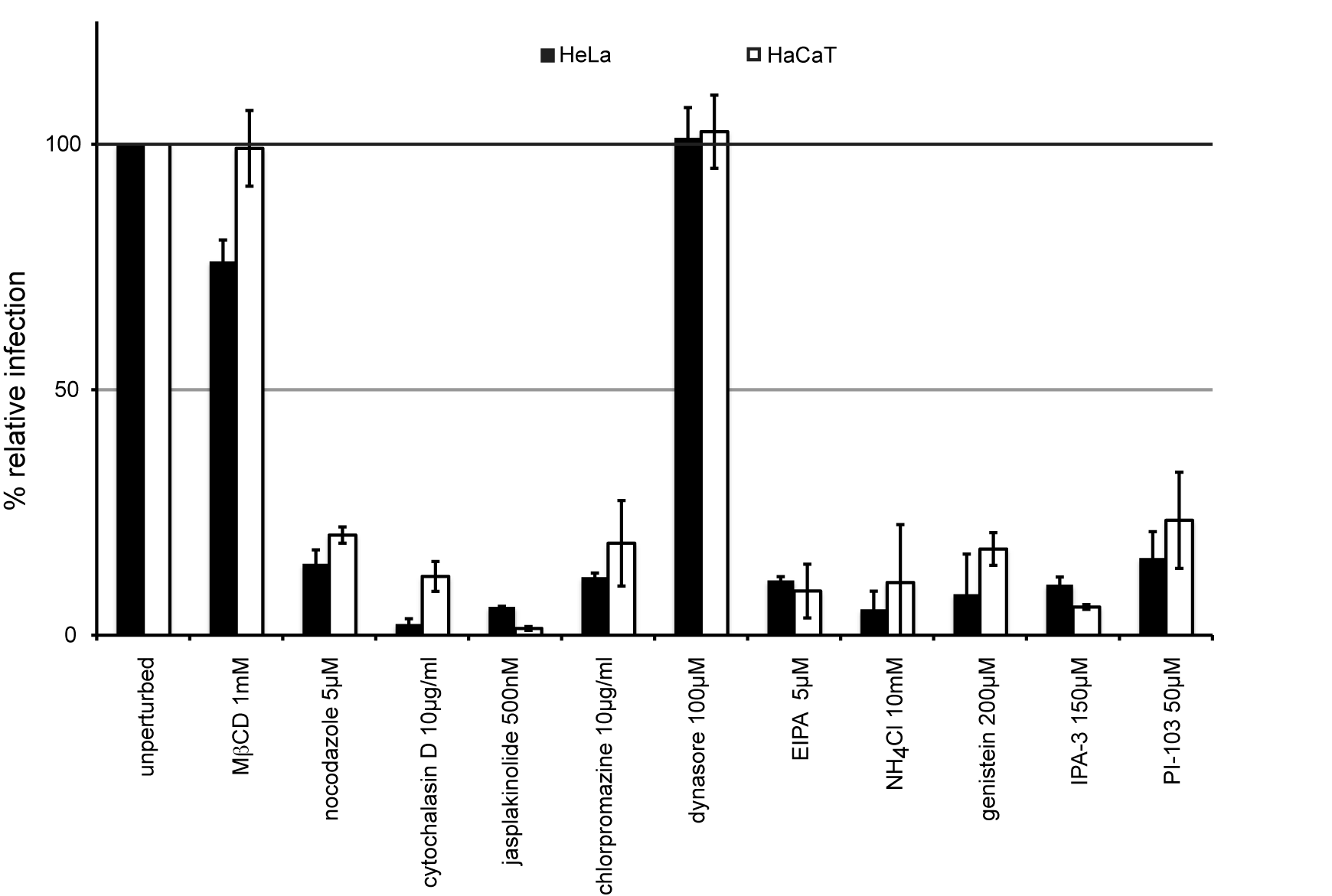

Supplement: Figure S4 — Cellular requirements for HPV-16 infection in HeLa vs. HaCaT cells. HeLa (black) and HaCaT (white) cells were pretreated with inhibitors at indicated concentrations for 30 min, and subsequently infected with HPV-16 PsV. 12 h p.i. inhibitors were exchanged for NH4Cl, and infection was continued for further 36 h. Cells were fixed and analyzed for GFP expression (infection) by flow cytometry, and infection is given relative to solvent treated control cells in %. Depicted are the results for three independent experiments ± SD. (TIF) [file ppat.1002657.s004.tif]

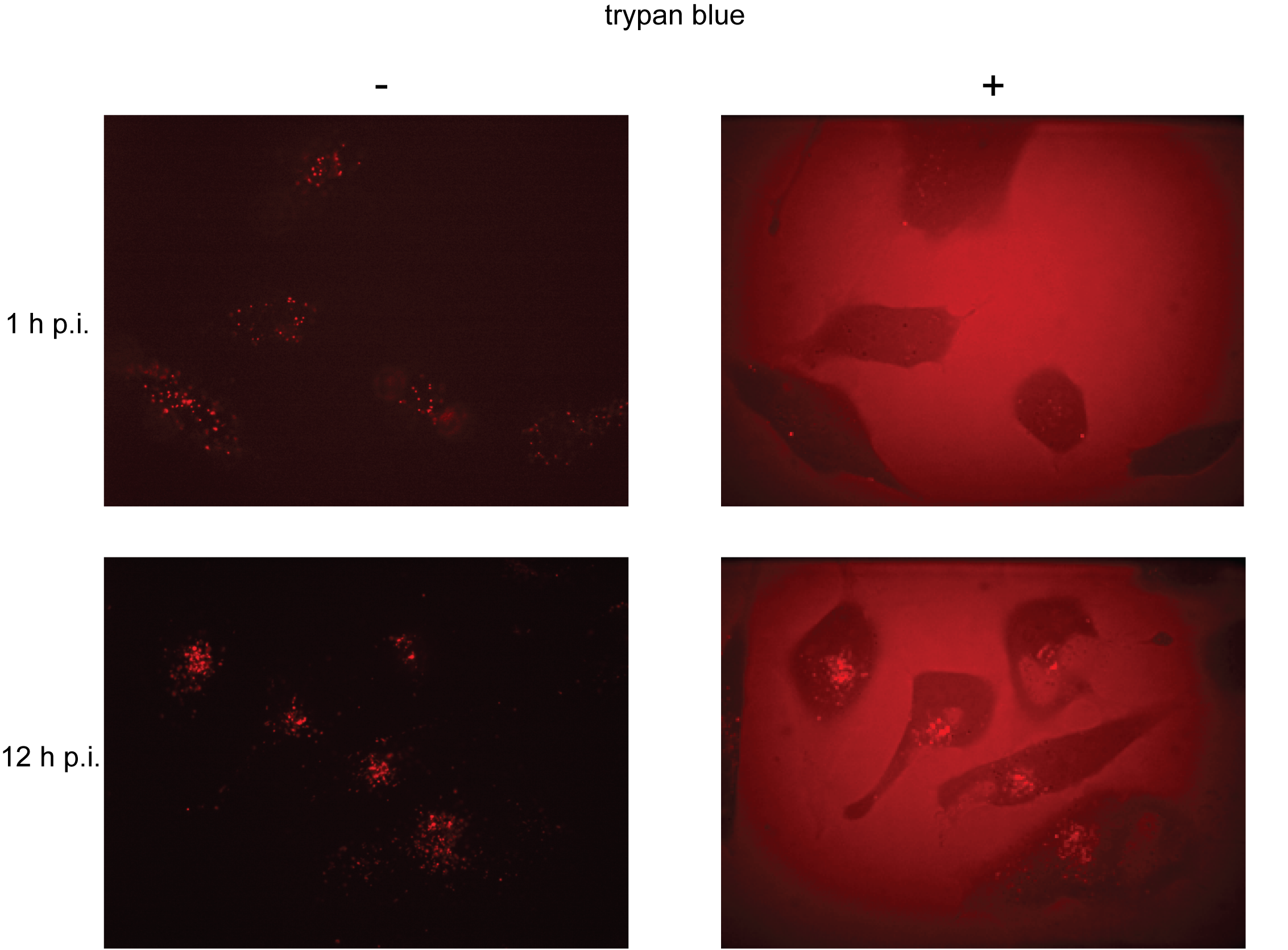

Supplement: Figure S5 — Trypan blue-based internalization assay of HPV-16 particles. HeLa cells were infected with AF594 labeled HPV-16 for 1 h (bound virus) or 12 h (internalized virus). Images of live cells are depicted before (−) and after (+) addition of trypan blue that quenches the fluorescence of externally located virus. (TIF) [file ppat.1002657.s005.tif]

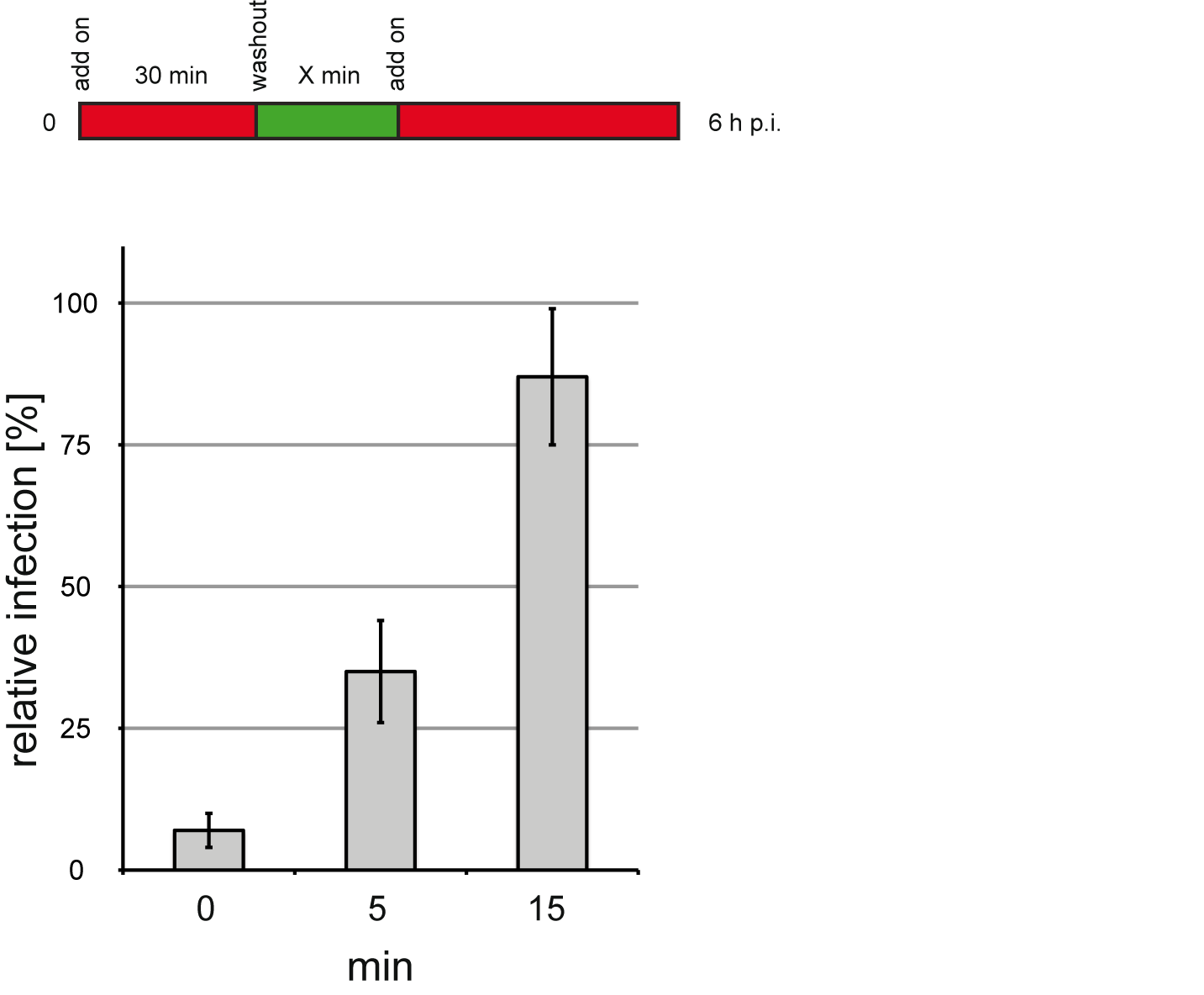

Supplement: Figure S6 — Kinetics if VSV acid activation in HeLa cells. HeLa were infected with VSV the presence of NH4Cl. 30 min p.i., the drug was washed out, and infection was continued in the absence of the drug for indicated times, after which NH4Cl was re-added and infection was continued in the presence of the drug, thus creating a time window of drug absence. Infection was scored 6 h p.i by flow cytometry. Depicted are results normalized to untreated control cells. (TIF) [file ppat.1002657.s006.tif]

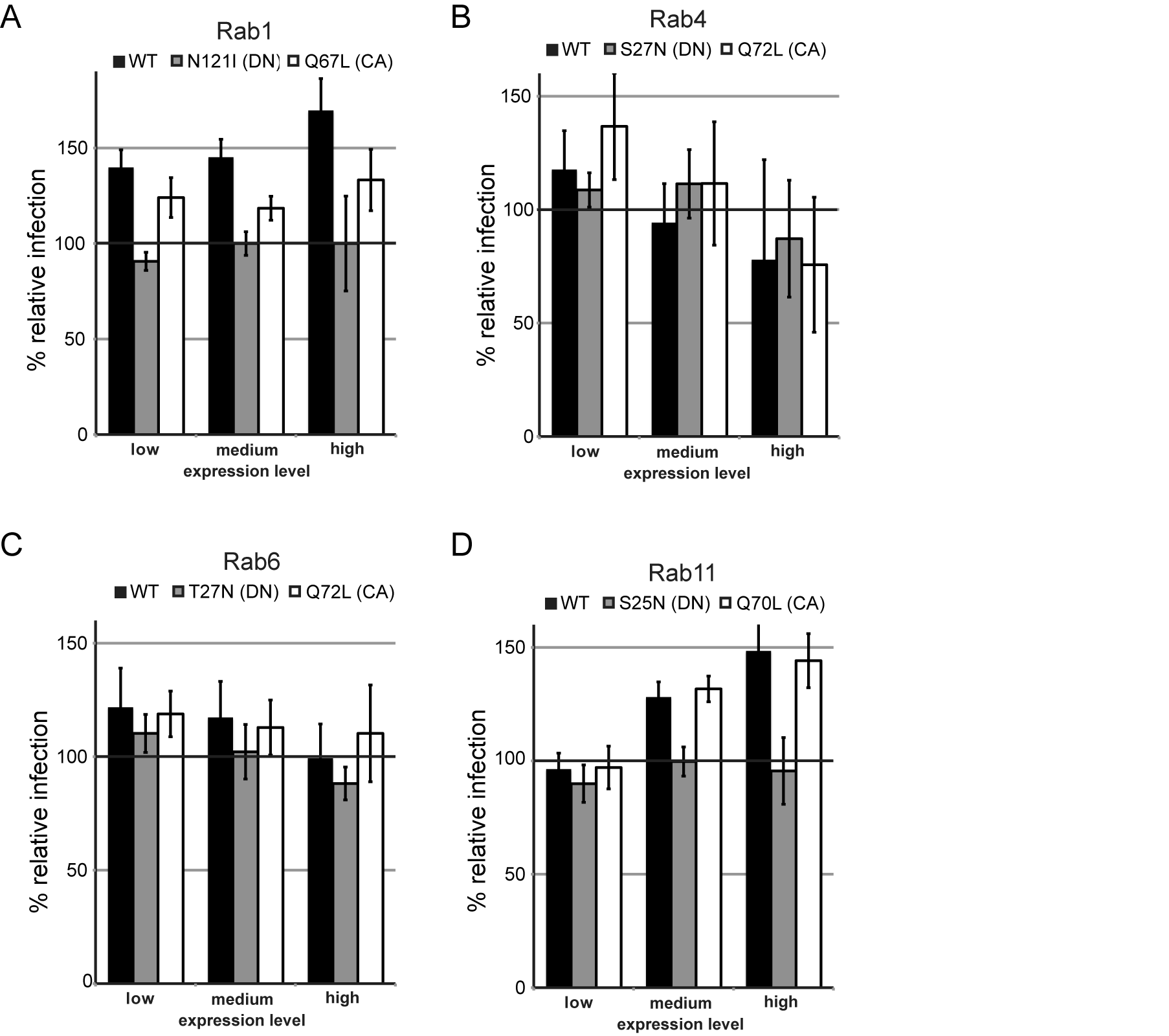

Supplement: Figure S7 — HPV-16 entry is Rab1, Rab4, Rab6, and Rab11-independent. HeLa cells were transfected with Rab1 (A), Rab4 (B), Rab6 (C), or Rab11 (D) fused to a GFP tag. Either the wildtype (WT, black), the DN (grey), or the CA (white) mutant were used. 24 h post transfection cells were infected with HPV-16 PsV expressing dsRed. Infection was scored by flow cytometry as in Figure 3A–C. Depicted are results normalized to GFP expressing control cells. (TIF) [file ppat.1002657.s007.tif]
