# Supplementary material for: Entry of Human Papillomavirus Type 16 by Actin-Dependent, Clathrin- and Lipid Raft-Independent Endocytosis
Source: PLoS Pathog. 2012 Apr 19;8(4):e1002657. doi: 10.1371/journal.ppat.1002657 (PMC3334892; doi:10.1371/journal.ppat.1002657)
Supplement: Table S1 — Additional infection data in the context of cell perturbations. The upper table summarizes data on virus infectivity after pharmacological inhibition of cellular targets. HeLa cells were pretreated for 30 min with pharmacological inhibitors in the indicated concentrations. Cells were infected with HPV-16 PsV, SFV, or SV40 in the presence of inhibitor as indicated. Inhibitors were exchanged for HPV-16 and SV40 with NH4Cl or DTT, respectively. Infection was scored by automated microscopy and image analysis. Depicted are results normalized to solvent treated control cells. See also Figure S2, and material and methods. The lower table summarizes data on virus infectivity after siRNA mediated depletion of cellular targets The efficiency of the knockdown is expressed as the amount of residual mRNA still present after siRNA transfection. HeLa cells were transfected with siRNA oligos directed against the indicated targets. Cells were infected with HPV-16 PsV and infection was scored 36 h p.i. by automated microscopy and image analysis. Depicted are the mean infection percentages relative to the the AllStar negative control. (PDF) [file ppat.1002657.s008.pdf]

**Target/Inhibitor**	**HPV-16**	**SFV**	**SV40**
	relative infection [%]		
Cholesterol/M-β-CD [mM]			
0,1	78±7	72±10	104±23
0,5	67±11	38±7	87±24
1	59±6	32±10	36±21
myosin II/blebbistatin [µM]			
10	84±8	74±25	115±50
25	87±14	107±4	85±31
50	73±19	93±23	75±8
MLCK/ML-7 [µM]			
2	100±22	127±14	112±37
10	122±22	103±16	110±13
50	78±50	118±53	79±14
PP1/Tautomycetin [µM]			
0,5	41±17	70±18	65±14
5	13±18	93±34	14±4
50	12±9	107±20	16±6
PKC/Rottlerin [µM]			
0,5	91±15	78±34	93±20
5	7±4	19±15	20±12
50	1±1	6±8	10±4
V-ATPase/Bafilomycin A1 [nM]			
1	67±20	87±30	96±9
10	2±1	17±12	56±4
100	1±1	8±5	19±2
Endosomal pH/Monensin [µM]			
1	17±9	21±8	69±24
10	2±1	9±4	49±17
30	2±2	19±10	48±16
Endosomal pH/NH_4Cl [mM]			
5	63±24	37±10	90±10
20	11±14	1±1	17±5
50	3±3	1±1	25±13

**siRNA**	**HPV-16**<br><br>**rel. inf. [%]**	**residual mRNA**<br>**% rel. to ctrl.**
**dynamin-2**		
Hs_DNM_8	90±25	17%
**Cdc42**		
Hs_CDC42_7	103±8	19%
**Rac1**		
Hs_RAC1_6	90+35	14%
**RhoA**		
Hs_RhoA_6	93±3	7%
**Arf6**		
Hs_ARF6_5	95±12	9%

Supplementary Table 1:
Cellular factors/processes tested for the involvement in HPV-16, SV40, SFV infection by pharmacological inhibition or siRNA knockdown
