# Supplementary material for: Entry of Human Papillomavirus Type 16 by Actin-Dependent, Clathrin- and Lipid Raft-Independent Endocytosis
Source: PLoS Pathog. 2012 Apr 19;8(4):e1002657. doi: 10.1371/journal.ppat.1002657 (PMC3334892; doi:10.1371/journal.ppat.1002657)
Supplement: Table S2 — List of the antibodies, pharmacological inhibitors, plasmids, siRNAs including their suppliers that were used in this study. (PDF) [file ppat.1002657.s009.pdf]

Antibodies/Stains

target	Supplier	Antibody/Product
**clathrin heavy chain**	BD Biosciences	610500
**AP2µ**	BD Biosciences	611350
**Actin**	Sigma	A3853
**caveolin-1**	Santa Cruz	sc-894
**flotillin-1**	BD Biosciences	610820
**flotillin-2**	BD Biosciences	610384
**EEA1**	BD Biosciences	610457
**LAMP-1**	Santa Cruz	sc-20041
**giantin**	Covance	PRB114C
**SFV E1, E2**		Singh and Helenius, 1992
**SV40 T-antigen**	G. Brandner (Freiburg)	Pab 1605
**IAV nucleoprotein**	Serotec	MCA400
**secondary antibodies**	Invitrogen	AF goat anti-mouse/rabbit antibodies, highly cross absorbed
**DNA/nucleus**	Invitrogen	H3569

Plasmids

name	Source	reference
**CLC-RFP**	James H Keen<br>(Thomas Jefferson University, USA)	Erdmann et al. 2007, Cell, 13:377-90
**Arf6 Q67L (DA)**	Urs F Greber<br>(University of Zürich, Switzerland)	Naslavsky et al. 2004, Mol Biol Cell. 15:3542-52
**Arf6 T27N (DN)**	Urs F Greber<br>(University of Zürich, Switzerland)	Naslavsky et al. 2004, Mol Biol Cell. 15:3542-52
**Arf6 wt**	Urs F Greber<br>(University of Zürich, Switzerland)	Naslavsky et al. 2004, Mol Biol Cell. 15:3542-52
**Cdc42 Q61L (DA)**	Ian Macara<br>(University of Virginia, USA)	Mercer et al. 2010, PNAS, 107(20):9346-51
**Cdc42 T17N (DN)**	Ian Macara<br>(University of Virginia, USA)	Mercer et al. 2010, PNAS, 107(20):9346-51
**Cdc42 wt**	Ian Macara<br>(University of Virginia, USA)	Mercer et al. 2010, PNAS, 107(20):9346-51
**dynamin-2 K44A (DN)**	Mark A McNiven<br>(University of Rochester, USA)	Cao et al. 1998, Mol Biol Cell. 9(9):2595-609.
**dynamin-2 wt**	Mark A McNiven<br>(University of Rochester, USA)	Cao et al. 1998, Mol Biol Cell. 9(9):2595-609.
**Rab1 N121I (DN)**	Cecilia Alvarez<br>(University of Alabama, USA)	Alvarez et al. 2003, Mol Biol Cell. 14:2116-27
**Rab1 Q67L (DA)**	Cecilia Alvarez<br>(University of Alabama, USA)	Alvarez et al. 2003, Mol Biol Cell. 14:2116-27
**Rab1 wt**	Cecilia Alvarez<br>(University of Alabama, USA)	Alvarez et al. 2003, Mol Biol Cell. 14:2116-27
**Rab11 Q70L (DA)**	Urs F Greber<br>(University of Zürich, Switzerland)	Wilcke et al. 2000, JCB. 151(6): 1207-20
**Rab11 S25N (DN)**	Urs F Greber<br>(University of Zürich, Switzerland)	Wilcke et al. 2000, JCB. 151(6): 1207-20
**Rab11 wt**	Urs F Greber<br>(University of Zürich, Switzerland)	Wilcke et al. 2000, JCB. 151(6): 1207-20
**Rab4 Q72L (DA)**	Urs F Greber<br>(University of Zürich, Switzerland)	McCaffrey et al. 2001, FEBS, 495:21-30
**Rab4 S27N (DN)**	Urs F Greber<br>(University of Zürich, Switzerland)	McCaffrey et al. 2001, FEBS, 495:21-30
**Rab4 wt**	Urs F Greber<br>(University of Zürich, Switzerland)	McCaffrey et al. 2001, FEBS, 495:21-30
**Rab5 Q79L (DA)**	Peter van der Sluijs<br>(Utrecht University School of Medicine, BEL)	Stenmark et al. 1995, Cell, 83: 423-32
**Rab5 S34N (DN)**	Peter van der Sluijs<br>(Utrecht University School of Medicine, BEL)	Stenmark et al. 1995, Cell, 83: 423-32
**Rab5 wt**	Peter van der Sluijs<br>(Utrecht University School of Medicine, BEL)	Stenmark et al. 1995, Cell, 83: 423-32
**Rab6 Q72L (DA)**	Urs F Greber<br>(University of Zürich, Switzerland)	White et al. 1999, JCB, 147 (4): 743-60
**Rab6 T27N (DN)**	Urs F Greber<br>(University of Zürich, Switzerland)	White et al. 1999, JCB, 147 (4): 743-60
**Rab6 wt**	Urs F Greber<br>(University of Zürich, Switzerland)	White et al. 1999, JCB, 147 (4): 743-60
**Rab7 S22N (DN)**	Urs F Greber<br>(University of Zürich, Switzerland)	Bucci et al. 2000, Mol Cell Biol, 11(2): 467-80
**Rab7 wt**	Urs F Greber<br>(University of Zürich, Switzerland)	Bucci et al. 2000, Mol Cell Biol, 11(2): 467-80
**Rac1 Q61L (DA)**	Ian Macara<br>(University of Virginia, USA)	Mercer et al. 2010, PNAS, 107(20):9346-51
**Rac1 T17N (DN)**	Ian Macara<br>(University of Virginia, USA)	Mercer et al. 2010, PNAS, 107(20):9346-51
**Rac1 wt**	Ian Macara<br>(University of Virginia, USA)	Mercer et al. 2010, PNAS, 107(20):9346-51
**RhoA G14V (DA)**	Ian Macara<br>(University of Virginia, USA)	Mercer et al. 2010, PNAS, 107(20):9346-51
**RhoA T19N (DN)**	Ian Macara<br>(University of Virginia, USA)	Mercer et al. 2010, PNAS, 107(20):9346-51
**RhoA wt**	Ian Macara<br>(University of Virginia, USA)	Mercer et al. 2010, PNAS, 107(20):9346-51

Inhibitors

Inhibitor	Product No.:	Supplier
**Bafilomycin A1**	B1793	SIGMA
**Blebbistatin**	B0560	SIGMA
**Chlostridium dif. C3 transferase**	CT04	Cytoskeleton
**Calphostin C**	C6303	SIGMA
**Chlorpromazine**	C8138	SIGMA
**Chlostridium dif. Toxin B**	C4102	SIGMA
**Cytochalasin D**	C8273	SIGMA
**Dynasore**	D7693	SIGMA
**EIPA**	A3085	SIGMA
**Genistein**	G6649	SIGMA
**H-7**	I6869	SIGMA
**IPA-3**	506106	Merck
**Jasplakinolide**	J7473	Invitogen
**Iressa**	G-4408	LC Laboratories
**Methyl-β-cyclodextrin**	C4555	SIGMA
**ML-7**	I2764	SIGMA
**Monensin A**	M5273	SIGMA
**NH_4Cl**	9718	Baker
**Nocodazole**	M1404	SIGMA
**Nystatin**	N6261	SIGMA
**Okadaic acid**	459616	Merck
**Orthovanadate**	567540	Merck
**PI-103**	52810	Merck
**Progesterone**	P8783	SIGMA
**Rottlerin**	R5648	SIGMA
**Wortmannin**	W1628	SIGMA

siRNAs

siRNA	Gene Symbol	Entrez Gene ID	mRNA accession	Gene Description	siRNA ID	target sequence
**AP2_Hin**	AP2M1	1173	NM_001025205, NM_004068	adaptor-related protein complex 2, mu 1 subunit		GAGCATGTGCACGCTGGCCAGCT
**AP2_Mot**	AP2M1	1173	NM_001025205, NM_004068	adaptor-related protein complex 2, mu 1 subunit		AAGTGGATGCCTTTCGGGTCA
**CHC_Hin**	CLTC	1213	NM_004859	clathrin, heavy chain (Hc)		AACCTGCGGTCTGGAGTCAAC
**Cav1_1**	CAV1	857	NM_001172895 NM_001172896 NM_001172897 NM_001753	Caveolin 1, caveolae protein, 22kDa	SI00299642	AAGCAAGTGTACGACGCGCAC
**Cav1_2**	CAV1	857	NM_001172895 NM_001172896 NM_001172897 NM_001753	Caveolin 1, caveolae protein, 22kDa	SI02654617	AAATTAAGAGCTTCCTGATTG
**CHC_Mot**	CLTC	1213	NM_004859	clathrin, heavy chain (Hc)		TAATCCAATTCGAAGACCAAT
**Flotillin-1**	FLOT1	10211	NM_005803	flotillin 1	SI00300237	AACAGATCCAGAGGATCTCTC
**Flotillin_2_1**	FLOT2	2319	NM_004475	flotillin 2	SI02781422	CATGCACATCATGTAGATTAA
**Flotillin_2_2**	FLOT2	2319	NM_004475	flotillin 2	SI02781457	CTGCCTGTCCCTTCTGGTAAA
**PAK1_1**	PAK1	5058	NM_002576, NM_001128620	p21 protein (Cdc42/Rac)-activated kinase 1	SI00605696	TCCACTGATTGCTGCAGCTAA
**PAK1_2**	PAK1	5058	NM_002576, NM_001128620	p21 protein (Cdc42/Rac)-activated kinase 1	SI00605703	TTGAAGAGAACTGCAACTGAA
**DNM_8**	DNM2	1785	NM_001005360, NM_001005361, NM_001005362, NM_004945, NM_001190716	dynamin 2	SI02654687	not disclosed
**CDC42_7**	CDC42	998	NM_001039802, NM_044472, NM_001791	cell division cycle 42 (GTP binding protein, 25kDa)	SI02757328	CATCAGATTTGAAATATTTAA
**RAC1_6**	RAC1	5879	NM_006908, NM_018890	ras-related C3 botulinum toxin substrate 1 (rho family, small GTP binding protein Rac1)	SI02655051	ATGCATTTCCTGGAGAATATA
**RhoA_6**	RHOA	387	NM_001664	ras homolog gene family, member A	SI02654211	TTCGGAATGATGAGCACACAA
**ARF6_5**	ARF6	382	NM_001663	ADP-ribosylation factor 6	SI02757286	CAACGTGGAGACGGTGACTTA

All siRNA duplexes from Qiagen.
